# Researching the Links Between Smartphone Behavior and Adolescent Well-being With the FUTURE-WP4 (Modeling the Future: Understanding the Impact of Technology on Adolescent’s Well-being Work Package 4) Project: Protocol for an Ecological Momentary Assessment Study

**DOI:** 10.2196/35984

**Published:** 2022-03-08

**Authors:** Steriani Elavsky, Jana Blahošová, Michaela Lebedíková, Michał Tkaczyk, Martin Tancos, Jaromír Plhák, Ondřej Sotolář, David Smahel

**Affiliations:** 1 Faculty of Informatics Masaryk University Brno Czech Republic

**Keywords:** well-being, adolescents, smartphones, intensive data, ecological momentary assessment

## Abstract

**Background:**

Smartphone ownership has increased among teens within the last decade, with up to 89% of adolescents owning a smartphone and engaging daily with the online world through it. Although the results of recent meta-analyses suggest that engaging digital technology plays only a small role in adolescent well-being, parents, professionals, and policymakers remain concerned about the impact that the instant connectivity of smartphones has on adolescent well-being.

**Objective:**

Herein, we introduce the protocol of a research study investigating the associations between adolescent smartphone use and different facets of well-being (social, physical, and psychological), with the aim to apply innovative methods to address the limitations of existing empirical studies.

**Methods:**

This 12-month prospective study of adolescents uses a repeated measurement-burst design with the ecological momentary assessment methodology. Adolescents (N=203; age range 13-17 years) complete baseline assessments through online questionnaires, four 14-day intensive data collection bursts, and an online questionnaire at the end of the study. As part of the 4 measurement bursts, adolescent smartphone behavior is assessed objectively by passive data collection of smartphone data logs and through self-reports in short questionnaires administered via a custom-built Android app.

**Results:**

The protocol describes the study objectives, research tools (including the development of the Android app and specialized software), and process (including pilot studies, the main study, and targets for machine learning approaches). Two of the 203 enrolled participants provided no data during the first data collection burst of the main study. Preliminary analyses of the data from the first data collection burst indicated an acceptable level of compliance (72.25%) with the daily questionnaires. The design of the study will allow for the assessment of both within- and between-person variabilities in smartphone behavior, as well as short-term variation and long-term change in smartphone behavior and how it impacts the indicators of social, physical, and psychological well-being.

**Conclusions:**

The innovative methods applied in this study (objective smartphone logs, ecological momentary assessment, and machine learning) will allow for a more nuanced assessment of the links between smartphone use and well-being, informing strategies to help adolescents navigate the online world more constructively in terms of the development of their physical, social, and psychological well-being.

**International Registered Report Identifier (IRRID):**

DERR1-10.2196/35984

## Introduction

### Background

Both socialization and leisure-time experiences of adolescents have largely shifted to the digital domain in the past decade. Smartphone ownership has increased among teens over the past 6 years, going from 41% in 2012 up to 89% today among 13- to 17-year-olds [1].

Over 80% of children aged 9 to 16 years report accessing the internet from their phones at least once daily, and only a minority report accessing the internet less often than daily or almost daily, ranging between 11% among Lithuanian children and 35% in France (average 20%) [2]. These European data are in line with data from the United States where close to 90% of adolescents now own smartphones and over 90% access the internet from it at least occasionally [3], suggesting that adolescents’ digital experiences occur mostly on their smartphones.

Smartphones, compared to other electronic media devices, enable instant connectivity to friends as well as the “online” world. Consequently, concerns have been raised about the impact this constant connectivity that smartphones afford could have on children’s mental health and well-being. These concerns have been stirred largely by findings from cross-sectional studies pointing to the links between time spent online (or intensity of mobile use) and various indicators of well-being. For example, studies have found links between being a heavy (versus light) user of digital technologies and lower well-being as well as having suicide risk factors such as depression [4-6]. However, the evidence from a study applying specification curve analysis across 3 large-scale data sets (total n=355,358) concluded that the association between digital technology use and adolescent well-being is negative but small, explaining at the most 0.4% of the variation in well-being [7]. On the other hand, a systematic review of 43 original research papers on adolescents concluded that information and communication technology usage can have benefits, such as in the form of higher self-esteem and higher perceived social support, although harmful effects were also reported, such as increased exposure to social isolation and depression [8]. The authors concluded that the majority of studies reported either mixed or no effects of technologies on adolescent well-being.

Still, a mostly negative discourse is prominent in the domain of physical well-being, where negative associations with technology have been demonstrated for sleep, physical activity, or overweight/obesity in children and adolescents. Namely, concerns over excessive screen time have been linked with low physical activity, poor sleep [9-12], and a higher risk of overweight/obesity rates [13]. This combined with the facts that levels of physical activity are already low among children and the proportion of overweight/obese children has been increasing in most Western populations has led to calls for public health guidelines and limits on children’s screen time, although the evidence to support them remains limited [14].

Regarding impacts on psychological or social well-being, both negative and positive associations with smartphone use have been identified. On the one hand, social network sites, which are now more accessible through smartphones, provide more opportunities for being in contact with other people, self-disclosure, and building intimate relationships, all of which are crucial for well-being [15]. On the other hand, using social network sites could be connected with negative outcomes when using these sites does not fulfill social needs such as the need for belonging or need for acceptance [16]. For example, a meta-analysis of 61 studies found a significant but weak relationship between time spent on social networking sites and depression and loneliness [17]. For lonely and socially anxious people, the use of social media is an easier way to be in touch with others [18]. However, they tend to use them excessively and more passively, which can paradoxically lead to declined well-being [19,20]. As such, it is clearly an issue in need of further exploration in order to properly educate young and vulnerable groups of people for promulgating beneficial patterns for social media use.

It is clear that the effects of mobile technologies are not uniform, with benefits conferred among some adolescents (eg, skill building among shy adolescents) and risks exacerbated among others (eg, worsening existing mental health problems). An increasing number of researchers are calling for studies that would be designed to capture the online experience more holistically and create a more nuanced picture of adolescent online experiences and their impacts [8,21]. Among the key limitations to existing studies are the relatively short duration of the studies and their reliance on self-reporting of smartphone behavior, despite related studies showing that people are poor judges of their online or smartphone use [22,23]. Longitudinal, experimental, and quasiexperimental studies that go beyond a reliance on self-reported information are required to understand how, for whom, and under what conditions adolescents’ interactions with mobile technologies influence their crucially developing social relationships, brains, and bodies. State-of-the-art approaches to managing online behaviors in children and adolescents will thus increasingly rely on methodologies incorporating objective data collection and artificial intelligence tools for the automatic detection of online risks and subsequent real-time interventions toward their mitigation.

While there are a number of opportunities associated with the deployment of technology-based data collection and innovative methodologies (eg, ecological momentary assessment [EMA]), there are also considerable risks associated with such research protocols. Among the advantages are the ability to separate between- and within-person variability, and closely examine the unfolding of temporal relationships between variables, making this approach good for theory testing. Other advantages are the ability to collect data from smartphones unobtrusively (eg, through mobile apps collecting data passively in the background), supplementing such data with sensor-based or self-reporting–based data without any recall bias allowing for a detailed granularity of data. However, such an approach is intrusive in terms of privacy for not only participants but also any third party that communicates with them, which means that special steps to ensure anonymity must be applied [24,25].

Herein, we introduce the protocol of the “Adolescents and Smartphone Use Study” that aims to investigate the associations between adolescent smartphone use and different facets of well-being (social, physical, and psychological) and, in doing so, implement innovations related to data-collection protocols so as to address some of the aforementioned empirical concerns. The study is part of the larger research project “Modelling the future: Understanding the impact of technology on adolescent’s well-being” (FUTURE [26]) that aims to develop a complex evidence-based theory depicting the impacts of technology usage on the physical, psychological, and social well-being of adolescents aged 11 to 18 years. This protocol describes the key elements of the research contained in one work package utilizing intense longitudinal data collection methods and innovative research tools with artificial intelligence.

### The FUTURE-WP4 Project

The overall aim of this project is to better assess the short-term and long-term impacts of smartphone use on well-being using innovative data collection approaches and the automatic recognition of adolescents’ online activities in real-time. Specifically, we planned to conduct a 12-month study of adolescent smartphone behavior that would utilize a repeated measurement-burst design [27] with 4 intensive 14-day data collection periods (ie, bursts) during which a specialized software (a custom-built mobile app) would capture numerous smartphone metrics and screenshots, and would distribute short questionnaires several times per day to assess aspects of subjective well-being and self-reported smartphone behavior. The project unfolded in several stages as presented below.

#### Stage 1: Evaluating the Ethical Aspects of the Study (2019)

During the first year of the study, we carefully evaluated the ethical and legal implications of the proposed work. An interdisciplinary team of experts was created to evaluate every aspect of the planned activities and create a risk/benefit analysis. Since the planned data collection was to include detailed records of adolescents’ online activities captured by a custom-made mobile app, we anticipated encountering numerous challenges associated with the ethics of such data collection, legal obstacles, and subsequent procedures for secure and careful data management. Therefore, the first phase of the project included a detailed examination of the possibilities for data collection and management, with the goal of creating a detailed study protocol that would comply with all ethical and legal standards. Team members who were experts in informatics, ethics, the social sciences, developmental psychology, and law met weekly for a year to discuss technology law and then the newly enacted General Data Protection Regulation policies, and to scrutinize the planned procedures, identify all problematic aspects, and help propose needed adjustments. During this phase, we also consulted with the Research Ethics Committee of Masaryk University. The output of these discussions was a final refined protocol for the subsequent processing of data collection and management deemed acceptable from ethical and legal perspectives (including the recommendation for developing a data anonymization tool, as described below). As a result of this process, the project received ethical approval from the university ethics board.

#### Stage 2: Software Development (2019-2020)

A fundamental aspect of the project was the development of a customized smartphone app for data collection. Designed to run in the background of the participants’ devices, the app passively captures key smartphone logs and screenshots during the active collection period. It also allows for the delivery of self-report questionnaires in a flexible manner (ie, based on different schemas, eg, timed, context-based, and self-initiated triggers). The collection parameters, such as start dates and participant groups, are organized with a companion web application for researchers. The software is backed by a dedicated server and a relational database. Additionally, as per the recommendation from the legal analysis process, we developed customized anonymization software.

##### Optical Character Recognition and the Anonymization Software

Optical character recognition (OCR) will be used to collect unstructured text from user screenshots, especially from instant messaging apps and web browsers. This way, we circumvent the inaccessibility of the data through the Android application programming interface (API) and potential ethical and legal issues by accessing the private space of the individual app’s storage. Additionally, it solves the extremely complicated task of anonymizing the screenshot images because only the extracted text will be saved, with the images themselves discarded.

Since such an automated data collection would also capture data from private (or at least nonpublic) conversations or profiles of people who had not provided consent with the study, it was necessary to develop a solution for automatic anonymization of the data during the data collection. Thus, we created software that would automatically anonymize the data during the collection and store only filtered anonymized data (ie, suppressing or masking the names, nicknames, addresses, and any other identifying information) [28].

##### Smartphone App for Data Collection and Questionnaires

We subsequently created an Android app (Interdisciplinary Research Team on Internet and Society [IRTIS] app) that became the primary building block for acquiring objective data from the participants. It collects various logs, such as screen activation, foreground apps, battery state, and connected Wi-Fi (for a complete list see Multimedia Appendix 1). The app also enables the delivery of questionnaires to get feedback from the participant or collect self-reported data on behavior and well-being. The app also allows the respondents to stop or pause the data collection at any time they wish. We enhanced it in the final stage of development by implementing a game-based reward system to improve the questionnaire compliance of the participants. Participants are familiarized with all features of the app (including the capturing of smartphone logs and screenshots) prior to the start of data collection.

We first released the app in November 2019. This release was focused on testing the overall function of the app on selected devices of volunteer testers. After the initial “in-house” testing phase, we tested the app more broadly through 3 pilot studies in the first half of 2020. The main focus of the pilot studies was to find and fix possible problems (especially related to screen data collection and the background running of apps) on various devices. We also introduced new features, such as messaging with our participants, new logs and their optimization to save battery life, dissemination of multiple data collection periods (referred to as “bursts”), and advanced management of questionnaires.

For the final pilot study at the end of 2020, we introduced the game-based reward system for enhancing questionnaire compliance and automated communication via messages with noncomplying participants. Participants could collect coins for completed questionnaires, which were then linked with lottery drawings for a number of prizes (eg, online vouchers). In this pilot, we also, for the first time, included questions intended for the main study, in order to test the general comprehension, reliability, and validity of the scales. The summary overview of the pilot tests and the details of the pilot testing process are described in Multimedia Appendix 2.

#### Stage 3: Machine Learning

One of the study goals is the development of predictive machine learning models for the automated detection, classification, and explanation of communicative behavior in the data collected from adolescents’ smartphones. Specifically, we focus on 2 types of communicative behaviors that can be associated with adolescents’ well-being (supportive online interactions and discussing risky behaviors online). We plan to use 2 supervised learning approaches. The first one uses structured data from the collected logs, and the other uses unstructured text from instant messenger (IM) conversations [29]. This work is in progress. We will use the data set with collected logs for the former, which is labeled by the time-matched answers in the self-reported questionnaires. The latter (IM conversations) requires schema development and manual annotation. The machine learning aspects of the project and both processes will be described in a separate manuscript.

## Methods

### Ethics Approval

This study was approved by the Research Ethics Committee of Masaryk University (EKV-2018-068).

### Design

The main study includes a 12-month prospective study of adolescents using EMA and a repeated measurement burst design. In EMA, participants are usually prompted several times a day to answer questions, and they may be asked to self-initiate a report when an event occurs, so as to capture phenomena as they unfold in natural environments in real life. This approach reduces the risk of retrospective recall bias associated with self-reporting or recollection of behavior [30]. EMA performed on smartphones has a number of advantages, including the automated recording of the timestamp of answers in real-time, the possibility of tracking of compliance and response patterns, and the possibility of combining survey responses with other data such as metrics from the smartphone device, from other online sources, or from a connected third device (eg, a sensor). This methodology also appears highly suitable for the study of adolescent online/smartphone behavior as it allows for a minimally obtrusive repeated assessment of authentic smartphone usage in the context of daily experiences, moods, and behaviors [31-33].

The study started in May 2021. At the beginning and end of the 12-month study, participants complete online surveys assessing key susceptibility variables and long-term well-being. Across the 12 months, participants complete 4 bursts of 2-week (10 weekdays and 4 weekend days) intensive data collection of smartphone behavior assessed passively through the custom-built Android mobile app (IRTIS app) installed on participants’ own smartphones, which should enhance the ecological validity of the collected data (as compared to relying on phones provided by a researcher). The app also administers short surveys 4 times per day, which assess real-time smartphone behavior and short-term changes (moment to moment, daily) in well-being. The surveys are administered in 4 predetermined time windows (6 AM-10 AM, 10 AM-3 PM, 3 PM-8 PM, and 8 PM-12 AM) on a semirandom schedule, with the exception of the morning survey. The morning survey had a default trigger time preset at 7 AM (to ensure it occurs before school), but the participants were encouraged to personalize this time with the possibility to select a different time between 6 AM and 10 AM that best fits their schedule (a different time could be set for each day of the week). All other surveys were triggered once at random within the respective time windows but always at least 1 hour apart. Upon notification, participants had 45 minutes to complete the morning and evening surveys and 90 minutes to complete the 2 daily surveys. The longer completion time for the daily surveys was chosen to ensure participants had a chance to complete the survey at the class break during school hours (a typical class period in the Czech Republic lasts 45 minutes, with 10- to 20-minute breaks in between) and to accommodate after-school activities. The surveys assess affective states, self-reported screen time, acute stressors, sexual content exposure, online vigilance, sporting and walking behaviors, and perceived social support (a list of items, item sources, and the EMA protocol can be found in Multimedia Appendix 3). The morning survey in addition has questions about sleep during the previous night. The evening survey also has retrospective questions about the day as a whole.

Additionally, participants are asked to complete a self-initiated report when something happened on the internet that left them bothered or upset (ie, uncomfortable discussions, news, pictures, or videos that left them feeling frightened or with an uneasiness afterward) through a self-initiated open-ended questionnaire. After each burst (on the 15th day), a summary postburst survey was administered. The postburst survey triggers at 7 AM, and participants have 12 hours to complete this survey.

The morning questionnaire and both daily questionnaires were designed to take under 2 to 3 minutes to complete, and the evening questionnaire was designed to take 4 to 5 minutes to complete. The actual duration of survey completion during the first burst was as follows: morning, median 77 seconds; daily I, median 24 seconds; daily II, median 23 seconds; evening, median 110 seconds; postburst, median 291 seconds.

### Participant Recruitment

The study sample was recruited from the Czech Republic with the help of a professional social science research and marketing company selected after a market review from multiple solicited bids. The specification was to find 300 adolescents aged 13 to 17 years, who have a smartphone with Android (at least version 5 “Lollipop”), and the sample was supposed to have an equal distribution across age and gender. Due to the lack of data from other comparable studies, the sample size was selected based on the pragmatic recommendation to recruit as many participants as we had resources for [34], while taking into account the rate of missing or problematic data from our pilot studies.

An independent social research and marketing company was commissioned to assist with study compliance maintenance. The research team managed most day-to-day responsibilities with real-time compliance monitoring during active data collection bursts. The company enforced compliance between bursts and handled problematic participants. The decision to involve a professional company in this way was partially motivated by the difficulty in offering incentives to participants from a university budget within the Czech legal framework.

### Materials and Procedures

**Figure 1:**
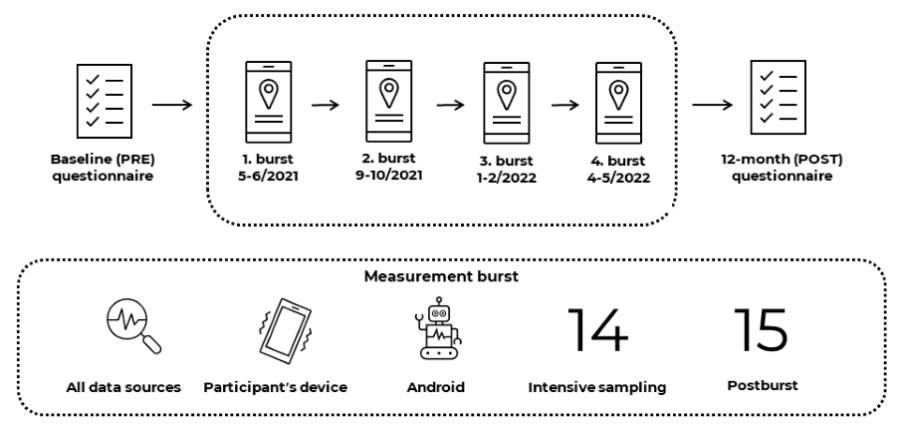
Depiction of the study design and protocol including the ecological momentary assessment bursts.

Upon recruitment, participants were asked to complete an online baseline questionnaire (administered via the Qualtrics platform). Subsequently, they were given instructions to install the study mobile app from the Google Play store. Participants were provided with a written manual with step-by-step instructions on downloading and operating the app and the study procedures. Short instructional videos were also created to facilitate the learning process. This strategy was chosen based on participant feedback from prior pilot studies. Personal demonstration or training was not feasible due to the ongoing COVID-19 pandemic. Each participant received their unique credentials to sign in to the app. The app automatically navigates the participant through permissions to collect different types of data upon the first sign-in. Researchers set up the survey assignment schedule for each of the 4 data collection bursts across the 12-month study in the researcher web application interface. The questionnaire schedule and 14-day bursts across the 12-month period are depicted in Figure 1.

### Participant Incentives

The participants receive no monetary compensation; however, they may win prizes in multiple lotteries, and the number of entries is determined by their questionnaire completion rate during the measurement burst. For each completed (at least 70% completed) questionnaire on a given day, the participant receives a bronze coin. When they complete 3 questionnaires (out of 4, or 75%), they receive a silver coin, and if they complete all 4 questionnaires, they receive a gold coin. A bronze coin is worth 1 lottery entry, a silver coin is worth 5 lottery entries, and a gold coin is worth 10 lottery entries. In total, 4 bronze coins, 1 silver coin, and 1 gold coin may be acquired in 1 day of a measurement burst, resulting in 19 entries. There is a lottery at the end of each measurement burst and at the end of the whole study. After each burst, participants may win vouchers to an electronics store in the amount ranging from 500 CZK to 2000 CZK (22 USD to 88 USD). In the final lottery at the end of the study, participants may win 1 of 2 smartphones (worth 7000 CZK and 10,000 CZK [209 USD and 442 USD, respectively]) or a Play Station 5 (13490 CZK [597 USD]).

### Research Hypotheses and Analytical Approach

Our main research questions center on the associations between smartphone use and its impact on different domains of well-being. We are especially interested in how different aspects of online behavior (assessed as patterns of smartphone use from the objective data logs) impact psychological, social, and physical well-being. Our design enables tracking of daily and momentary short-term fluctuations in well-being indicators as well as long-term change (across four bursts) and how it is influenced by patterns of smartphone app use (eg, time spent in social networking apps, mobile games, communication apps, or browsers). We are also interested in the reciprocal relationship between smartphone usage and well-being indicators so as to obtain a more nuanced view of the temporal associations between the 2 (eg, through examination of cross-lagged effects). Given the hierarchical nature of the data (moments nested within days, bursts, and persons), we plan to utilize multilevel approaches including vector autoregressive models to capture associations among change over time and to disentangle the between-person and within-person associations among outcomes of interest.

## Results

### Sample Description

**Figure 2:**
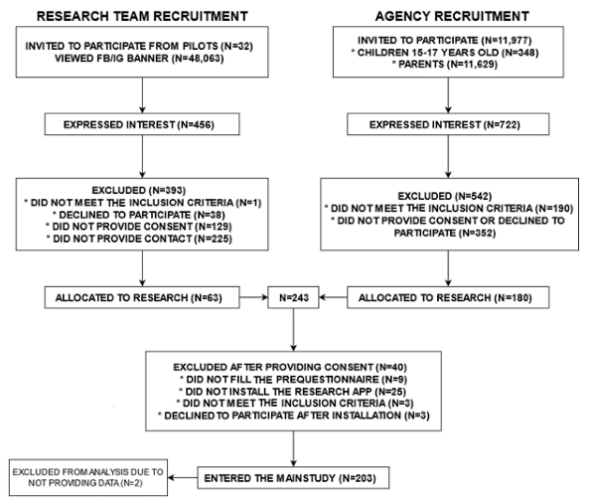
CONSORT (Consolidated Standards of Reporting Trials) diagram describing participant recruitment. FB: Facebook; IG: Instagram.

In total, 203 adolescents were enrolled in the study. The social science research and marketing company recruited participants through their network of adult and adolescent respondents. However, given the unique type of research that requires intensive participation and poses a serious privacy intrusion, the agency was not able to find a sufficient number of participants (see CONSORT [Consolidated Standards of Reporting Trials] diagram in Figure 2). The agency invited almost 12,000 children or parents with children; however, only 180 participants provided informed consent. Thus, we supplemented the sample with participants from our own recruitment efforts through paid advertisements on social media and through chain referral, which yielded an additional 63 participants. In the end, overall, 243 participants provided informed consent, but only 203 completed all requirements (ie, provided consent [both the participant and their parent], completed the baseline questionnaire, and installed the app) and entered the study. Two participants did not provide any data (daily questionnaire data and metrics data) during the first measurement burst. A descriptive overview of the final study sample can be seen in Table 1.

**Table 1:** Study sample.

Age group (years)	MEDIAN agency		IRTIS^a^ team	
	Girls (n=54), n	Boys (n=89), n	Girls (n=30), n	Boys (n=30), n
13	15	25	2	0
14	13	24	0	4
15	11	21	2	5
16	12	8	9	7
17	3	11	17	14

^a^IRTIS: Interdisciplinary Research Team on Internet and Society.

### Preliminary Compliance

Data collection for the first burst took place in 2 waves (2 weeks apart). The second wave was added to accommodate the schedules of some participants and the stragglers (n=21) who submitted their informed consent and baseline questionnaire after the deadline. Each wave installed the app on Thursday and started data collection on Friday. The overall completion rate of the timed (morning, daily I, daily II, and evening) questionnaires was 72.3% (6690 completed surveys [with at least one answered question] out of 9260 notified surveys). The completion rate was higher for school days (80.4%; 5356 completed surveys out of 6662 notified surveys) than for weekend days (68.13%; 1770 completed surveys out of 2598 notified surveys). There was some failure in delivering surveys (related to notifications). Despite planning for each burst to present 56 surveys (4 surveys × 14 days) for an expected collection total of 11,256 surveys, only 9260 (82.3%) were successfully notified on the participants’ smartphones. The completion rate for each type of survey (as well as the corresponding “success” rate for the actually delivered surveys) is included in Table 2.

**Table 2:** Survey completion rates for the first burst.

Survey	Number of completed surveys (at least one answered question)	Number of notified surveys	Completion rate (number of completed surveys/number of notified surveys)	Successful delivery rate (number of notified surveys/number of planned surveys)
Morning	1613	2319	69.56% (1613/2319)	82.41% (2319/2814)
Daily I	1696	2320	73.10% (1696/2320)	82.44% (2320/2814)
Daily II	1802	2336	77.14% (1802/2336)	83.01% (2336/2814)
Evening	1579	2285	69.10% (1579/2285)	81.20% (2285/2814)
Postburst^a^	149	165	90.30% (149/165)	82.09% (165/201)
Self-initiated	21	N/A^b^	N/A	N/A

^a^Values calculated based on n=201 (ie, without 2 participants who did not provide any data).

^b^N/A: not applicable.

## Discussion

This paper introduces the protocol of a study focusing on the smartphone behavior of adolescents and its impact on physical, psychological, and social well-being. In our study, we combine passive data collection of smartphone logs with the intensive assessment of self-reported states and behaviors through a mobile app. The design of our study (a prospective measurement-burst design) will allow us to capture both short-term variability in outcomes of interest as well as long-term change. We apply the EMA methodology, which has been shown to be feasible and productive in studies of adolescents [31,35]. When supplemented with our app, this methodology allows us to link self-reported data with data from objective smartphone logs, affording the analysis of the temporality of effects at both the within- and between-person levels. Concretely, this means being able to compare the impact of different levels and patterns of smartphone use (eg, heavy versus light usage) on well-being, as well as assessing how changes in smartphone behavior over time reflect on well-being (eg, whether spending more time online on one’s smartphone than usual leads to increased or decreased well-being).

Collecting smartphone log data is a strength of our approach. This allows for the collection of objective data with minimum demands on study participants (ie, data are collected automatically and passively in the background). However, studies from other fields suggest that when it comes to behavior prediction, there may be a trade-off between accuracy or data details and participant burden. For example, in studies of dietary behavior, prediction models using collected sensor-based data (posing minimum participant burden) have resulted in lower accuracy than models using self-reported data through EMA prompts when predicting dietary lapses [36]. The extent to which smartphone log data will successfully predict behavior or well-being will likely depend on the operationalization of the objective log data. For example, when assessing smartphone behavior, one must take into account an entire spectrum of behaviors that may have different impacts. A user may passively scroll the news feed or engage actively with other people in the comments section. In each case, we may expect a different impact on user psychological outcomes [37]. Thus, different “metrics” must be generated from the smartphone log data to capture these different aspects of behavior.

Some behaviors may also be more difficult to operationalize using smartphone log data. Consider, for example, the question of how to accurately capture what exactly adolescents are doing when spending time in a specific social networking app. While we may be able to effectively quantify the time spent in social networking apps, it is more challenging to capture what happens during the social interactions while on the app. Moreover, this may be crucial when evaluating the association between app use and psychological and social well-being [38]. For example, a study using momentary sampling techniques showed that individuals who are involved in a greater amount of supportive interactions with others feel more positive emotions after these interactions and report more perceived social support [39]. While we are unable to directly evaluate the “quality” of online interactions, the OCR tool we are developing for the analyses of screenshots from mobile phones along with the application of machine learning algorithms could lead to a more nuanced assessment of adolescent smartphone behavior in future research. Other limitations associated with the smartphone log data include constraints applied directly by Google Play policies, which preclude the collection of some data (eg, GPS and web-browsing histories). The use of web browsers may result in exposure to very different types of content (eg, educational and harmful), thus leading to different types of psychological outcomes.

A critical component to interpreting the data from our study will be the consideration of selection bias and how the resulting sample differs from the general adolescent population of smartphone users. While the study was not planned to be representative, it will be key to understand in what ways our participants differ from the general population. The recruitment process posed a big challenge in spite of the extensive experience that the IRTIS team has with large-scale studies of children and adolescents. We attribute this primarily to the sensitive nature of the data collected and the perceived intrusiveness into the privacy of the adolescents. Despite carefully considering the ethical implications of the data collection, creating detailed descriptions of data handling and safety procedures (for both parents and children), developing anonymization software, and having received the approval of relevant ethical bodies, some participants reported distrust and intrusion of privacy among the reasons for nonparticipation. The social research and marketing agency tasked with the recruitment of participants in our study provided feedback and recommendations for the future, including offering financial incentives to all participants (not only through lottery drawings), extending the inclusion criteria to iOS users, and further alleviating privacy concerns through modifications in the research design.

The development of the custom mobile app spanned a period of 2 years, and in spite of extensive pilot testing, we were unable to fully eliminate technical issues associated with data collection. For example, there was a 17.74% failure rate of surveys not being properly delivered to the users’ devices (only 9425 surveys out of 11,457 planned surveys were properly notified). This is a problem primarily from the standpoint of inflating missing data. Even when a survey notification is activated, it is common for users to not notice the notification or simply disregard it. While certain strategies can help enhance response rates (eg, incentives and gamification [40]), the lack of responses due to technical problems/failure should be minimized. We found that problems of failed notifications were more common in certain types of devices (especially the smartphones of some manufacturers, such as Huawei and Xiaomi). Additionally, ongoing optimization of the Android system and its updates necessitate ongoing technical and programming support throughout all phases of the study. Our app was downloadable through Google Play, where we also faced privacy policy limitations on data collection and therefore had to modify our app throughout the pilot testing phase (eg, deleting the GPS location component).

Although there were some challenges with the application of EMA (eg, adapting the EMA protocol to fit youths’ school schedules and complications related to the ongoing COVID-19 pandemic such that we had to incorporate additional questions in the postburst survey regarding school attendance/distance learning), data from the first burst indicated acceptable levels of compliance with the study protocol. The average compliance rate in the first burst in our study was in line with other EMA studies of adolescents where compliance varies widely from 51.56% to 92.00% in studies of psychological outcomes [41] or 43.8% to 95.9% in studies of health behaviors [42]. In a meta-analysis of EMA studies targeting children and adolescents [43], the compliance rate in studies prompting participants 4 to 5 times per day was 77.4%, which is only slightly higher than in our study (72.25%). The data are however not directly comparable, since the majority of studies included in the meta-analysis were short-term (did not plan to involve more than one data collection burst) or used research devices that were novel for users, perhaps inflating compliance estimates. In our study, we relied on participants’ own devices. As is recommended in EMA studies [31,44], we continually monitored study adherence with a system of both automatic notifications and reminders (in-app), as well as telephone, SMS, and email check-ins and follow-up contacts when necessary. We also provided compliance-based incentives to support participant engagement. Additional strategies are being considered to boost adherence in follow-up bursts (eg, bolstering contact with participants in-between bursts through activities such as a Christmas competition).

The challenges surrounding accurate data collection related to real-time smartphone usage remain, especially in light of continual technological or software innovation and the ever-morphing proclivities of user engagement. As such, it demands increased vigilance relative to the processes by which research in this realm is conducted. Studies that seek to employ innovative, nuanced, and more comprehensive approaches to the protocol of collecting the said data are, therefore, of paramount importance. Further refinement of research instruments, protocols, and methodologies is needed to obtain a more accurate portrait of how adolescents are actually engaging with their online worlds. This was and remains our goal, as we seek to develop, produce, and implement procedures that will more effectively assist adolescents to navigate these worlds more constructively in terms of the development of their physical, social, and psychological well-being.

## Appendix

Multimedia Appendix 1 Objective smartphone data codebook.

Multimedia Appendix 2 Pilot studies in the Adolescents and Smartphone Use Study.

Multimedia Appendix 3 Ecological momentary assessment study protocol for the Adolescents and Smartphone Use Study.

Multimedia Appendix 4 Peer-review report by the Czech Science Foundation.

## Abbreviations

API: application programming interface
EMA: ecological momentary assessment
IM: instant messenger
IRTIS: Interdisciplinary Research Team on Internet and Society
OCR: optical character recognition
