# Supplementary material for: Researching the Links Between Smartphone Behavior and Adolescent Well-being With the FUTURE-WP4 (Modeling the Future: Understanding the Impact of Technology on Adolescent’s Well-being Work Package 4) Project: Protocol for an Ecological Momentary Assessment Study
Source: JMIR Res Protoc. 2022 Mar 8;11(3):e35984. doi: 10.2196/35984 (PMC8941440; doi:10.2196/35984)
Supplement: Multimedia Appendix 1 [file resprot_v11i3e35984_app1.docx]

# Multimedia Appendix 1. Objective smartphone data codebook.

Note: **Limit** means the maximum time (in seconds) between two records in the database so that we can consider them as two consecutive records between which there was no failure (outage). For example, if I have a timestamp of a record of 8:00:00 and a limit of 300 seconds, then the next record is taken as a follow-up if it has a timestamp of 8:05:00 (on the same day) and earlier. **Currently, all limits are set to 210 seconds.**

**Link to issues:** <https://gitlab.fi.muni.cz/irtis/irtis-statistic-generator/-/issues>

The transcription.csv table is now automatically generated from current data on Google Play.

Collection intervals according to Michal's documentation

(https://docs.google.com/document/d/1qthL_SwILgtLzdMNFyMyP5hwDW7D4bk2vTEuto2h8Ms/edit#heading=h.2vge8mgtcxz5):

| **Metric** | **Period** | **Values** | **Manual** | **Optimization** |
| --- | --- | --- | --- | --- |
| Screenshots | 5 | Device screen image, metadata | Yes | Yes |
| ~~Location~~ | ~~5~~ | ~~Latitude, longitude~~ | No | Yes |
| Activity | 5 | Name of a type of an activity | No | Yes |
| Steps | ? | Count of steps | ? | ? |
| Battery | 2 | Battery state percents | No | Yes |
| ~~Wifi available~~ | ~~60~~ | ~~Name~~ | No | Yes |
| ~~Wifi connected~~ | ~~30~~ | ~~Name~~ | No | Yes |
| ~~Calls~~ | ~~10m~~ | ~~Number, time, text, type~~ | ~~No~~ | ~~No~~ |
| ~~Sms~~ | ~~10m~~ | ~~Number, time, text, type~~ | ~~No~~ | ~~No~~ |
| Application | 1 | Name (both foreground and background apps), runtime | No | No |
| Notifications | ? | Count, source | ? | ? |
| Screen | 1 | on/off | No | No |
| Screen tap | 1 | on/off | No | No |
| Headphones | 2 | on/off | No | No |
| Playback | 1 | on/off | No | No |

## User_id

Identification number of the user.

## Dat

Date (day) for which given statistics are being calculated.

## Metrics_on_time

Limit = 210

The amount of time metrics have been turned on. It is calculated from a “screen” table keeping records of whether or not the screen has been turned on. Records should be saved every second without optimization, but due to lagging (probably internal optimization of the android itself on some phones), the spacing of records may be larger. However, this happens in the vast majority of cases when the screen is turned off (probably every time, but not verified). Both True (screen on) and False (screen off) entries are counted.

## Metrics_on_per_day

Limit = 210

It is calculated the same as Metrics_on_time, but is given here as a percentage. 50% means that the collection of metrics took place through half of the day, i.e. 12 hours.

## Screen_on_time

Limit = 210

The amount of time the screen has been on. It is calculated from the table “screen” (see Metrics_on_time), however this time only records with a value of True are considered.

For maximum accuracy, the time between True and False records is calculated for this metric. Specifically, the time difference between two True records is taken in full (if it is within the time limit), for False True (or False True) records only half of the time difference is taken (only if the entire difference is within the time limit).

Screen_on_time_0-6

The amount of time the screen has been on between midnight and 6 am (i.e., 0:00:00 - 5:59:59).

Screen_on_time_6-12

The amount of time the screen has been on between 6 am and noon (i.e., 6:00:00 - 11:59:59).

Screen_on_time_12-18

The amount of time the screen has been on between noon and 6 pm (i.e., 12:00:00 - 17:59:59).

Screen_on_time_18-22

The amount of time the screen has been on between 6 pm and 10 pm (i.e., 18:00:00 - 21:59:59).

Screen_on_time_22-24

The amount of time the screen has been on between 10 pm and midnight (i.e., 22:00:00 - 23:59:59).

## Screen_on_of_count

Limit = 210

The number of times the screen was turned on after it was turned off. It is calculated from the table “screen” (see Metrics_on_time) and we consider the “turn-on event” as the True record that is preceded by any number of non-zero False records.

## Screen_on_off_leq_5

The number of screen “turn-on events” that lasted 5 seconds or less.

## Screen_on_off_leq_30

The number of screen turns that lasted a maximum of 30 seconds.

## Screen_on_of_geq_600

The number of screen turns that lasted at least ten minutes (600 seconds).

## Location_per_day

Limit = 210

The amount of time GPS position data has been collected. The time between two records is calculated if their difference is within the time limit. It is given as a percentage of total time collecting smartphone log data per day.

## Battery_per_day

Limit = 210

The amount of time battery data has been collected. The time between two records is calculated if their difference is within the time limit. It is given as a percentage of total time collecting smartphone log data per day.

Wifi_per_day

Limit = 210

The amount of time wifi connection data has been collected. The time between two records is calculated if their difference is within the time limit. It is given as a percentage of total time collecting smartphone log data per day.

Wifi_per_screen_on

Limit = 210

The amount of time that wifi connection data was collected. Unlike Wifi_per_day, it only takes into account records that were collected when the screen was turned on. The time between two such records is calculated if their difference is within the time limit. It is expressed as a percentage of the time the screen was turned on that day.

## Screenshots_time

Limit = 210

The amount of time during which screenshots were taken. The time between two records is calculated if their difference is within the time limit.

## Screenshots_per_screen_on

The amount of time the screenshots were taken divided by the time the screen was turned on. That is, it is the value “100 * Screenshots_time / Screen_on_time” in %.

Note Screenshots should only be saved when the screen is on. However, in the current data, they are taken even more often (poor detection of the screen lock). Therefore, the value is over 100%. It would be possible to calculate similarly to Wifi_per_screen_on, but I see no reason for that (the correction will occur on the application side and this way we can at least detect a possible problem).

## Playback_time

Limit = 210

The amount of time during which data on time when music (sound) was played was collected. The time between two records is calculated if their difference is within the time limit.

## Steps

Limit = 210

The number of steps the user has taken based on the internal counter.

Note: If there are two records on different days, then the number of steps is divided evenly into both days.

Note: Some phones display the number of steps cumulatively, so the counter may be very inaccurate on the first day.

## Activities - STILL, ON_FOOT, WALKING, RUNNING, IN_VEHICLE, ON_BICYCLE, TILTING, UNKNOWN

Limit = 210

The time during which the individual activities were collected. The time between two records is calculated if their difference is within the time limit. If two consecutive records contain the same activity, the entire time difference is added up for that particular activity. If two consecutive records (within the time limit) contain different activities, then half of the time difference of the record is credited to the first and half to the second activity.

Note: The “WALKING” and “RUNNING” activities are subcategories of the “ON_FOOT” activity.

Note: The “WALKING” and “RUNNING” activities are not currently collected correctly - the system does not supply them. We are working on getting them from another place where they should be recorded correctly.

## Applications (including Screen taps) - Youtube, Instagram

Limit = 210

The record consists of several values:

0:44:31 (27.19%) - 22 - 616

launch time (percentage of time the screen was on) - the number of times the application was turned on for at least 3 seconds - the number of screen touches while in the application

**The time that individual applications have been running:** We only consider records that were collected when the screen was turned on. The time between two records is calculated if their difference is within the time limit. If two consecutive records contain the same application, the entire time difference is added up for that application. If two records in a row (within the time limit) contain different applications, then half of the time difference of the record is credited to the first and half to the second application.

The header is taken from a transcription table, which is created on the basis of the title as it appears in Google Play (see, for example, [School online](https://play.google.com/store/apps/details?hl=en_US&id=cz.skolaonline.mobile)) - if there is a transcript, it can be entered in the header. If the record does not exist in Google Play, then the program tries to find the translation in the manually filled-in table transcription_manual_in.csv. If the transcript does not exist in it either, then the original package name will be used.

**The number of times the application is turned on** determines how many times the application has been turned on for at least 3 seconds. The time limit is applied here to filter out unwanted events (e.g., the application will remain running when the screen is off and will appear for a second after turning screen on before the user switches to another application or when it may switch to the foreground for a while when switching between applications).

For each application, **the number of screen touches** that occurred when the application was in the foreground is listed.

Note For example, if you wanted to create some categories, this would theoretically be possible via the transcription.csv table.

Note: Only one application in the foreground is running at one time and we collect data about it.

## App_time_sum

The sum of the times in the applications that are listed.

Note: There may be applications that are not in the list (and yet the user had them turned on that day) and their time is not listed in the table and therefore not included in the total sum.

## App_time-Screen_on_time

App_time_sum value minus Screen_on_time value. Thus, a negative number means that a longer screen-on time has been calculated compared to the sum of applications.

## Drop_outs

Outages where no metrics were collected. The individual records are separated by a semicolon. These are always intervals - the first time determines the time of the last record (in the format hour: minute) and the second determines the time when the next record was obtained (in the same format), while the time difference is greater than the limit for the metric Metrics_on_time

## What other tables do we have

**ApplicationBackground**

**Notification**

**Runtime**
