# Supplementary material for: Researching the Links Between Smartphone Behavior and Adolescent Well-being With the FUTURE-WP4 (Modeling the Future: Understanding the Impact of Technology on Adolescent’s Well-being Work Package 4) Project: Protocol for an Ecological Momentary Assessment Study
Source: JMIR Res Protoc. 2022 Mar 8;11(3):e35984. doi: 10.2196/35984 (PMC8941440; doi:10.2196/35984)
Supplement: Multimedia Appendix 2 [file resprot_v11i3e35984_app2.docx]

# **Multimedia Appendix 2. Pilot studies in the Adolescents and Smartphone Use Study.**

The software development and testing unfolded through numerous iterations of internal testing and across five pilot studies with participants from the target population. All five pilot studies included samples of children from the target population (ages 13-17 and Android users) and tested the functionality of the custom-built app across 14 days, including two weekends. Additionally, all pilot studies participants were also asked for their Messenger conversations dating back to one year. This was optional, and the conversations were downloaded by the participants directly from Messenger and delivered via a desktop app that stripped the data of any images and videos. The conversations, after anonymization software was applied, served as a basis for the machine learning component.

The table below summarizes key information related to the pilot studies.

|  | Pilot 1 | Pilot 2 | Pilot 3 | Pilot 4 | Pilot 5 |
| --- | --- | --- | --- | --- | --- |
| Duration | 27. 3. – 4. 4.2020 | 5. – 29. 5.2020 | 12. – 26. 6.2020 | 26.11. – 10.12.2021 | 3. – 17. 12.2021 |
| Recrutation | Via leaflets sent to schools in Czech Republic, mostly through chain referral | Via banners posted in Facebook groups targeted adolescents, parents or educators | Via paid Facebook advertisement targeted children aged 13-17 years | Via paid Facebook advertisement targeted children aged 13-17 years | Via paid Facebook advertisement targeted children aged 13-17 years |
| Participants | N = 4 (1 girl, 3 boys) | N = 7 (5 girls, 2 boys) | N = 34 (14 girls, 20 boys) | N = 31 (14 girls, 17 boys) | N = 8 (6 girls, 2 boys) |
| Age | 13-17 years,  M = 14.5, SD = 1.3 | 13-15 years,  M = 13.6, SD = 0.7 | 13-17 years,  M = 15.8, SD = 1 | 13-17 years,  M = 16.1, SD = 0.8 | 13-18 years,  M = 14.7, SD = 1.8 |
| EMA prompts | Short surveys twice per day (morning, evening) | Short surveys twice per day (morning, evening) | 4 surveys per day, pseudo-random schedule (four time blocks: 8:00-12:00, 12:00-16:00, 16:00-20:00, 20:00-22:00) | Surveys 4 times per day, pseudo-random schedule (one survey in four time blocks: 6:00-10:00, 10:00-15:00, 15:00-20:00, 20:00-22:00) | |
| Tested components | Testing of smartphone metrics data collection and survey functionality | Testing of smartphone metrics data collection and survey functionality | Testing of four survey time blocks per a day and gamification motivating component | Final version of the application and survey items were tested, simulating a measument burst from the main study | |
| Reward for participation | All participant received 32 GB USB stick | All participants received 32 GB USB stick, chance to win a game hosting coupon (3x500 CZK) or electronics shop coupon (2x1000 CZK) | | All respondents had chance to win 32 GB USB stick (10 pieces) and Electronics shop coupon (5x1000 CZK) | |
