# Supplementary material for: Researching the Links Between Smartphone Behavior and Adolescent Well-being With the FUTURE-WP4 (Modeling the Future: Understanding the Impact of Technology on Adolescent’s Well-being Work Package 4) Project: Protocol for an Ecological Momentary Assessment Study
Source: JMIR Res Protoc. 2022 Mar 8;11(3):e35984. doi: 10.2196/35984 (PMC8941440; doi:10.2196/35984)
Supplement: Multimedia Appendix 3 [file resprot_v11i3e35984_app3.docx]

# **Multimedia Appendix 3. Ecological momentary assessment study protocol for the Adolescents and Smartphone Use Study.**

**Participants:** N = 203

**Inclusion criteria:** aged 13-17, owns smartphone with Android (min. version 5.0 or higher), connecting to the internet regularly via Wi-Fi

**Design:** 1-year study with a measurement burst design (4 x 14 days with 3 months apart)

**EMA protocol:** 4 x day pseudo-random survey; plus self-initiated report of uncomfortable internet experience; plus 1 post burst survey after each 14-days period; objective smartphone use data logs, screenshots collection

Maximal number of timed surveys per person = 4 x 14 x 4 = 224 + 4 post burst surveys

**EMA surveys:**

1. Timed trigger – 4 x day with a 90min buffer; morning survey at a concrete chosen time in a predefined time window, other three surveys randomly in predefined time windows
2. 6:00-10:00 (must be completed in 45 minutes)
3. 10:00-15:00 (must be completed in 90 minutes)
4. 15:00-20:00 (must be completed in 90 minutes)
5. 20:00-00:00 (must be completed in 45 minutes)

The survey has a notification reminder every 10 minutes and can be snoozed for 10 minutes. The survey is sent to the server when it is completed or after the time for completion expires (eve when not completed). Notification windows for the morning and evening surveys can be adjusted to adapt to individual wake or bedtimes. Morning survey can be set at a concrete time, or it is notified at 7 am by default. Evening survey can be set to notify until concrete bedtime.

1. Self-initiated trigger (uncomfortable internet experience report) – a participant may report any episode when they experienced something that bothered them on the internet, what made them feel uncomfortable or scared. It could include annoying discussions on social media, news, pictures, or videos that they came across or that someone sent them.
2. Post burst survey – day after the 14day burst period, one longer survey summarizing the last fourteen days is notified in application. It is notified at 7 am and available for completion for 12hours (between 6:00 and 18:00).

**EMA training:**

Participants were provided with a written manual with a step-by-step instruction on downloading and operating the app and the study procedures. Additionally, five short instructional videos were also created to facilitate the learning process. This strategy was chosen based on participant feedback from prior pilot studies.

**I. TIMED SURVEYS**

**Morning (6:00-10:00) items 1. to 4., 6., 7., 10. to 18.**

**Daily Surveys I. And II. (10:00-15:00, 15:00-20:00) items 5., 10. to 18.**

**Evening (20:00-00:00) items 8. to 34.**

| **Item Order** | **Variable** | **Item** | **Response Format** | **Logic** |
| --- | --- | --- | --- | --- |
| **Sleep** | | |  |  |
| **1.** | Sleep_1A | What time did you go to bed yesterday? Indicate in hours and minutes format, e.g.,22:15. | HOD:MIN (00:00) |  |
| **2.** | Sleep4 | How long did it take for you to fall asleep last night? | HOD:MIN (00:00) |  |
| **3.** | Sleep2_A | What time did you wake up this morning? Indicate in hours and minutes format, e.g. 06:15. | HOD:MIN (00:00) |  |
| **4.** | *Sleep6_A | How many hours and minutes did you actually sleep last night? (This may differ from the number of hours spent in bed) | HOD:MIN (00:00) |  |
| **5.** | Sleep3 | How would you rate your sleep quality last night? | (0) “Very bad” – (100) “Very good” |  |
| **6.** | Sleep5 | Do you feel drowsy right now? | (0)“Not at all“ – (100) „Very“ |  |
| **Technology** |  |  |  |  |
| **7.** | Tech1_A | How much time did you spend last night (from 8 pm until you fell asleep) using screens EXCEPT your mobile phone, e.g., PC, laptop, or TV? Indicate in hours and minutes format, e.g.,2.5 hours, enter 02:30. | HOD:MIN (00:00) |  |
| **8.** | Tech2_A | How much time did you use ICTs, e.g., mobile phone, computer, laptop, tablet in school during the day (until this questionnaire)? Indicate using hours and minutes format, e.g.,4.5 hours, enter 04:30. | HOD:MIN (00:00) |  |
| **9.** | Tech3_A | How much time have you spent today, EXCEPT school teaching, together with EXCEPT mobile phone technologies, eg on a desktop computer, laptop or television? Enter hours and minutes, e.g., 2.5 hours, enter 02:30. | HOD:MIN (00:00) |  |
| **Smartphone use** | | | | |
| **10.** | **Smartphone_use_1 | For how long were you using your smartphone (including phone calls, being online, playing games, listening to music, etc.) during the day (up to this questionnaire). Enter the hours and minutes (e.g., enter 5.5 hours as 05:30). | HOD:MIN (00:00) |  |
| **11.** | **Smartphone_use_2 | How many times did you check your smartphone during the day? Write down the number of times you've done it. | HOD:MIN (00:00) |  |
| **Affect (random selection of four items – always one of Affect1-3, Affect4-6, Affect7-9, Affect10-12)** | | | | |
| *How are you feeling right now?* | | | *Slider with word anchors* |  |
| **11.** | Affect1 | Calm | (0) “Not at all” – (100) “Very” |  |
| **11.** | Affect2 | Content | (0) “Not at all” – (100) “Very” |  |
| **11.** | Affect3 | Relaxed | (0) “Not at all” – (100) “Very” |  |
| **12.** | Affect4 | Sad | (0) “Not at all” – (100) “Very” |  |
| **12.** | Affect5 | Lonely | (0) “Not at all” – (100) “Very” |  |
| **12.** | Affect6 | Bored | (0) “Not at all” – (100) “Very” |  |
| **13.** | Affect7 | Energetic | (0) “Not at all” – (100) “Very” |  |
| **13.** | Affect8 | Happy | (0) “Not at all” – (100) “Very” |  |
| **13.** | Affect9 | Excited | (0) “Not at all” – (100) “Very” |  |
| **14.** | Affect10 | Nervous | (0) “Not at all” – (100) “Very” |  |
| **14.** | Affect11 | Upset | (0) “Not at all” – (100) “Very” |  |
| **14.** | Affect12 | Angry | (0) “Not at all” – (100) “Very” |  |
| **Daily stressors** | | |  |  |
| **15.** | Dise | In the past hour, have you been bothered or stressed by these situations? (Select all that concern you) | *(1) Problem with family*  *(2) Problem with friends or classmates*  *(3) Problem with school*  *(4) I feel stressed because of something else*  *(5) I was not stressed* |  |
| **16.** | Dise1 | Problem with family | (0) “Not at all” – (100) “Very” | IF Dise=1 |
| **17.** | Dise2 | Problems with friends or classmates | (0) “Not at all” – (100) “Very” | IF Dise=2 |
| **18.** | Dise3 | Problem with school | (0) “Not at all” – (100) “Very” | IF Dise=3 |
| **19.** | Dise4 | I feel stressed because of something else | (0) “Not at all” – (100) “Very” | IF Dise=4 |
| **Sexual exposure material** | | |  |  |
| **20.** | Sex_A | Have you seen any sexual pictures, images or videos (i.e., depicting naked people or people having sex) since last evening? | (0) No  (1) Yes  (2) Prefer not to say |  |
| **21.** | Sex1_A | Did you want to see these images or videos? | (1) Yes  (2) Rather yes  (3) Rather no  (4) No  (5) Prefer not to say | IF Sex=1 |
| **22.** | Sex2_A | How did you feel about seeing such images? | (1) Very happy  (2) Rather happy  (3) Neither happy nor upset  (4) Rather upset  (5) Very upset  (6) Prefer not to say | IF Sex=1 |
| **Online vigilance and salience** | | |  |  |
| **23.** | OV_S_bin | In the last half an hour, how much were you thinking about something that happened on the internet or other online activities? | (0) No (1) Yes |  |
| **24.** | OV_S_A | How much were you thinking about these activities? | (0) “Not at all” – (100) “Very” | IF OV_S_bin = (1) |
| **25.** | OV_V_A | How did you perceive these thoughts about online events or activities? | (0) “Very uncomfortable” – (100) “Very comfortable” | IF OV_S_bin = (1) |
| **Sport and walk** | | |  |  |
| **26.** | Sport1_A | Did you do any sports activities today besides physical education at school? | (0) No  (1) Yes |  |
| **27.** | Sport2_A | How much time did you spend on sports activities today (up until the time of this survey), EXCEPT for physical education at school? Enter hours and minutes, e.g., enter 45 minutes as 00:45. | HOD:MIN (00:00) | IF Sport1=1 |
| **28.** | Walk1_A | How much time (until this questionnaire) did you spend by walking outside today? Indicate in hours and minutes format, e. g. 45 min, enter 00:45. | HOD:MIN (00:00) |  |
| **Perceived social support** | | |  |  |
| *How much do you agree / disagree with the following sentence? After today, I feel like I have friends or classmates around me who …* | | | *Slider with word anchors* |  |
| **29.** | PS_INF | …advise me when i need it | (0) “Not at all” – (100) “Very” |  |
| **30.** | PS_EM | …understands my feelings | (0) “Not at all” – (100) “Very” |  |
| **31.** | PS_B | …ask me to join activities | (0) “Not at all” – (100) “Very” |  |
| **32.** | PS_ES | …appreciate what I do | (0) “Not at all” – (100) “Very” |  |
| **33.** | PS_INS | …shares his or her things with me when I need it | (0) “Not at all” – (100) “Very” |  |

**II.SELF-INITIATED INTERNET RISK REPORT**

| **Item order#** | **Variable** | **Item** | **Response Format** | **Logic** |
| --- | --- | --- | --- | --- |
| *Click on this questionnaire if something happened on the Internet that left you bothered or upset* | | |  |  |
| **1.** | Risks_self_initiated | You said something happened to you on the internet that left you bothered or upset (you felt uncomfortable or frightened). Please describe, what happened to you on the internet. | Text |  |

**III. POSTBURST SURVEY**

| **Item Order#** | **Variable** | **Item** | **Response Format** | | | **Logic** | | | | |
| --- | --- | --- | --- | --- | --- | --- | --- | --- | --- | --- |
| **Typical days** | | |  |  | | | | | | |
| **1.** | Days_typical | In this questionnaire we will ask you about THE LAST 14 DAYS. The last 14 days have been typical for me. | (1) Strongly disagree,  (2) Disagree,  (3) Slightly disagree,  (4) Neither disagree nor agree,  (5) Slightly agree, (6) Agree,  (7) Strongly agree | | | | | |  | |
| **2.** | Mobile_main | The smartphone on which I fill out this questionnaire is my primary smartphone, which I use most often during the day. | (1) Yes, (2) No | | | | | |  | |
| **3.** | Phone_behavior_typical | The following questions will concern your primary smartphone, which you use most often. For THE LAST 14 DAYS, I've been using my smartphone in a typical way. | (1) Strongly disagree,  (2) Disagree,  (3) Slightly disagree,  (4) Neither disagree nor agree,  (5) Slightly agree, (6) Agree,  (7) Strongly agree | | | | | |  | |
| **4.** | Phone_behavior_atypical | Over the last 14 days, I have changed the way I use my smartphone in a significant way due to my participation in the research. | (1) Strongly disagree,  (2) Disagree,  (3) Slightly disagree,  (4) Neither disagree nor agree,  (5) Slightly agree,  (6) Agree,  (7) Strongly agree | | | | | |  | |
| **5.** | Covid_school_short_A | How many days have you been to school in the last 14 days? By that, we mean your physical presence at school. Enter the number of days from 0 to 10. | 0, 1, 2, 3, 4, 5, 6, 7, 8, 9, 10 | | | | | |  | |
| **6.** | Covid_school_2_A | How many days have you attended online learning in the last 14 days? Enter the number of days from 0 to 10. | 0, 1, 2, 3, 4, 5, 6, 7, 8, 9, 10 | | | | | |  | |
| **7.** | Covid_school_3 | If you had online learning, which device did you use most often for attending it? | (1) Laptop,  (2) Desk computer (PC),  (3) Smartphone,  (4) Tablet,  (5) Other | if covid_school_2 = 1,2,3,4,5,  6,7,8,9,10 > covid_school_3 | | | | | | |
| **Restrictive parental behaviour** | | |  |  | | | | | | |
| **8.** | Restrict_yn | We will now ask a few questions concerning your parents' control over how you use your smartphone. Over the past 14 days, my parents have somehow limited my time spent on my smartphone, such as total time per day or using the phone in the evening. | (1) Yes, (2) No | |  | | | | | |
| **9.** | Restrict_app | For the past 14 days, I've had an app running on my smartphone that limits the time I spend with it. | (1) Yes, (2) No | |  | | | | | |
| **10.** | Restrict_time_spent_A | How much time have you been allowed to use your smartphone during a day in the last 14 days? | (1) Unlimited  (2) No more than 1 hour  (3) No more than 2 hours  (4) No more than 3 hours  (5) No more than 4 hours  (6) No more than 5 or more hours per day | |  | | | | | |
| **11.** | Restrict_evening_A | Have you had any restrictions on the use of your smartphone in the EVENING in the last 14 days or has your phone been blocked by an application? | (1) Yes, (2) No | |  | | | | | |
| **12.** | Restrict_evening_2 | If you have restrictions on your smartphone, please indicate the time from which you may not use it in the evening, or it is blocked by the application running on your phone. | HOD:MIN (00:00) | If restrict_evening=1 >  restrict_evening_2 | | | | | | |
| **Excessive internet use** | | |  |  | | | | | | |
| *In the past 14 days, how often it happened to you…* | | |  | | | |  | | | |
| **13.** | Excessive_use_1_A | That you have gone without eating or sleeping because of the internet? | (1) Never  (2) Almost never  (3) On some days  (4) About half of these days  (5) Most days or every day | | | |  | | | |
| **14.** | Excessive_use_2_A | That you have felt bothered when I cannot be on the internet? | (1) Never  (2) Almost never  (3) On some days  (4) About half of these days  (5) Most days or every day | | | |  | | | |
| **15.** | Excessive_use_3_A | That you have caught yourself using the Internet although I’m not really interested | (1) Never  (2) Almost never  (3) On some days  (4) About half of these days  (5) Most days or every day | | | |  | | | |
| **16.** | Excessive_use_4_A | That you have spent less time than you should with either family, friends or doing schoolwork because of the time I spent on the internet? | (1) Never  (2) Almost never  (3) On some days  (4) About half of these days  (5) Most days or every day | | | |  | | | |
| **17.** | Excessive_use_5_A | That you have tried unsuccessfully to spend less time on the internet? | (1) Never  (2) Almost never  (3) On some days  (4) About half of these days  (5) Most days or every day | | | |  | | | |
| **Bedroom mobile use and sleep** | | |  | | | |  | | | |
| **18.** | *Napping | We will now ask you questions concerning your sleep. The following questions are about your usual sleep habits. How often during the last 14 days did you take a nap (fell asleep during the day)? | (1) Never  (2) Almost never  (3) On some days  (4) About half of these days  (5) Most days or every day | | | | | | |  |
| **19.** | Bedroom_screens | How long before sleep do you usually stop using all technologies and devices, i.e., mobile phones, tablets, computers etc.? For example, if you put down the phone and then go to bed right away, you should indicate 0 hours and 0 minutes. | HOD:MIN (00:00) | | | | | | |  |
| **20.** | Bedroom_mobile | Where do you usually put your smartphone while you sleep? | (1) In my immediate vicinity (e.g., next to bed),  (2) On the other place in my room,  (3) In other room than the one I sleep in | | | | | | |  |
| **21.** | Bedroom_mobile_regime | What smartphone profile do you usually set when you sleep? | (1) switched off,  (2) switched on but in silent profile, i.e. with no sound and no vibration,  (3) switched on in regular profile, with sound or vibration | | | | | | |  |
| **22.** | Insomnia_1 | Please indicate how often it happened to you in the last 14 days that you had difficulty falling asleep | (1) Never  (2) Almost never  (3) On some days  (4) About half of these days  (5) Most days or every day | | | | |  | | |
| **23.** | Insomnia_2 | Please indicate how often it happened to you in the last 14 days that you had difficulty staying asleep | (1) Never  (2) Almost never  (3) On some days  (4) About half of these days  (5) Most days or every day | | | | |  | | |
| **24.** | Insomnia_3 | Please indicate how often it happened to you in the last 14 days that you had problems waking up too early | (1) Never  (2) Almost never  (3) On some days  (4) About half of these days  (5) Most days or every day | | | | |  | | |
| **25.** | Insomnia_4 | Please indicate how often it happened to you in the last 14 days that your sleep problems interfered with your daily functioning (e.g., daytime fatigue, mood, ability to function at school, etc.)? | (1) Never  (2) Almost never  (3) On some days  (4) About half of these days  (5) Most days or every day | | | | |  | | |
| **Smartphone use** | | |  | | |  | | | | |
| **26.** | **Smartphone_use_1 | In the last 14 days…ON A SCHOOL DAY: how long were you using your smartphone (including phone calls, being online, playing games, listening to music, etc.)? Enter the hours and minutes (e.g., enter 5.5 hours as 05:30). | HOD:MIN (00:00) | | |  | | | | |
| **27.** | **Smartphone_use_2 | In the last 14 days…ON A WEEKEND DAY: how long you were using your smartphone (including phone calls, being online, playing games, listening to music, etc.)? Enter the hours and minutes. | HOD:MIN (00:00) | | |  | | | | |
| **28.** | **Smartphone_use_3 | In the last 14 days…ON A SCHOOL DAY: how much time did you spend using social networking and communication apps (e.g., Instagram, Facebook, TikTok, Messenger, WhatsApp) on your mobile? Enter the hours and minutes. | HOD:MIN (00:00) | | |  | | | | |
| **29.** | **Smartphone_use_4 | In the last 14 days…ON A WEEKEND DAY: how much time did you spend using social networking and communication apps (e.g., Instagram, Facebook, TikTok, Messenger, WhatsApp) on your mobile? Enter the hours and minutes. | HOD:MIN (00:00) | | |  | | | | |
| **30.** | **Smartphone_use_5 | In the last 14 days…ON A SCHOOL DAY: how much time did you spend playing games on your smartphone? Enter the hours and minutes. | HOD:MIN (00:00) | | |  | | | | |
| **31.** | **Smartphone_use_6 | In the last 14 days…ON A WEEKEND DAY: how much time did you spend playing games on your smartphone? Enter the hours and minutes. | HOD:MIN (00:00) | | |  | | | | |
| **32.** | **Smartphone_use_7 | In the last 14 days…ON A SCHOOL DAY: how much time did you spend watching YouTube and Twich videos on your smartphone? Enter the hours and minutes. | HOD:MIN (00:00) | | |  | | | | |
| **33.** | **Smartphone_use_8 | In the last 14 days…ON A WEEKEND DAY: how much time did you spend watching YouTube and Twich videos on your smartphone? Enter the hours and minutes. | HOD:MIN (00:00) | | |  | | | | |
| **34.** | **Smartphone_use_9 | In the last 14 days…ON A SCHOOL DAY: how many times did you check your smartphone (i.e., briefly turned it on or unlocked the screen)? Write down the number of times you've done it. | NUMERIC | | |  | | | | |
| **35.** | **Smartphone_use_10 | In the last 14 days…ON A WEEKEND DAY: how many times did you check your smartphone (i.e., briefly turned it on or unlocked the screen)? Write down the number of times you've done it. | NUMERIC | | |  | | | | |
| ***question added from 2^nd^ burst on; **question added from 3^rd^ burst on** | | | | | | | | | | |


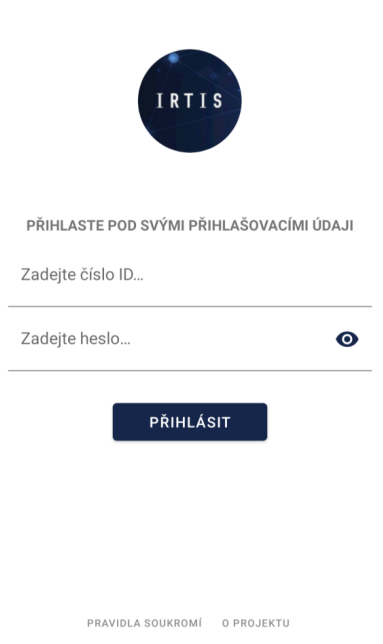

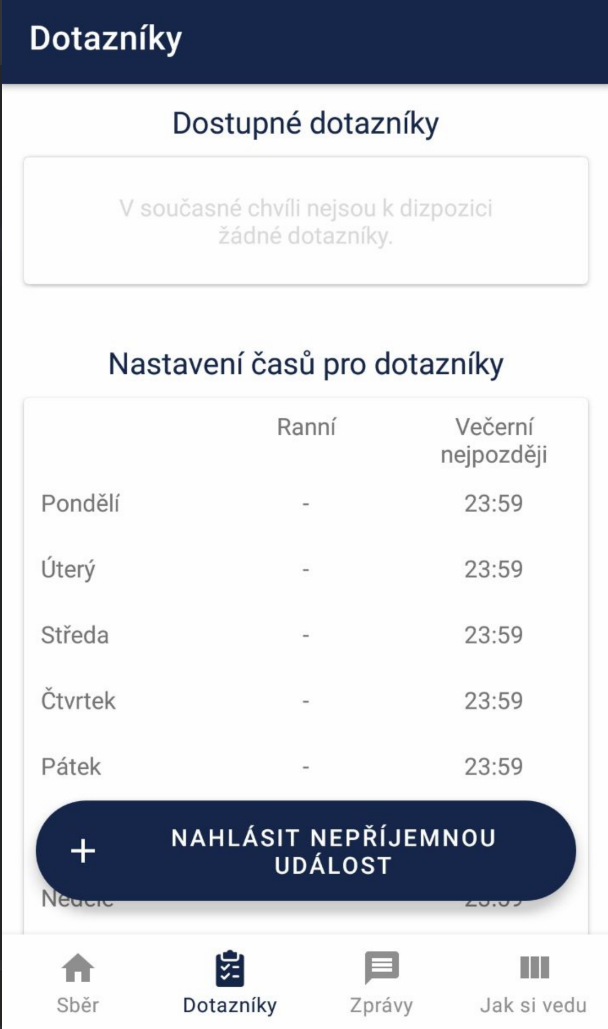


English caption:

Surveys

Available surveys

Currently no surveys are available

Time setting for surveys

Morning Evening

Monday

Tuesday

Wednesday

Thursday

Friday

Saturday

Sunday

+ Report uncomfortable experience

Burst|Surveys|Messages|My Progress

English caption:

Sing-in under your login details.

Enter your ID number

Enter your password

Sign-in


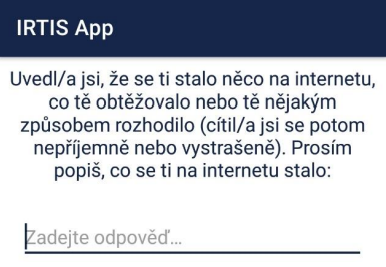


English caption:

IRTIS App

You said something happened to you on the internet that left you bothered or upset (you felt uncomfortable or frightened). Please describe, what happened to you on the internet?

Enter the answer


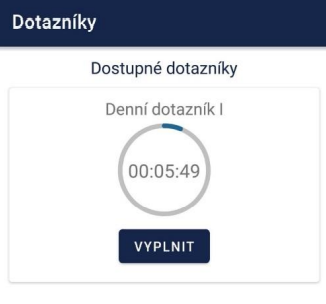


English caption:

Surveys

Available surveys

Daily survey I

Expires for 5:49

Fill in


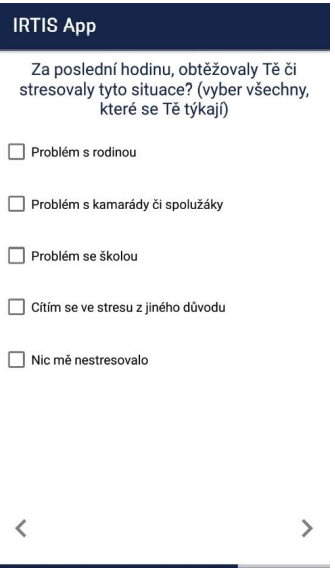


English caption:

IRTIS App

In the past hour, have you been bothered or stressed by these situations? (Select all that concern you)

- Problem with family
- Problem with friends or classmates
- Problem with school
- I feel stressed because of something else
- Nothing stressed me


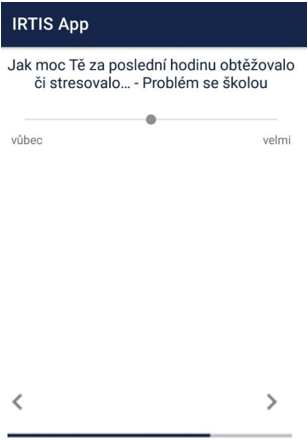

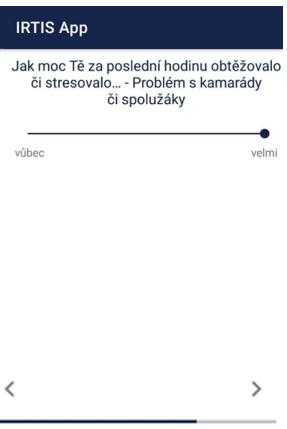


English caption:

IRTIS App

In the past hour, have you been bothered or stressed by… - Problem with friends or classmates

Not at all---------------------------------Very

English caption:

IRTIS App

In the past hour, have you been bothered or stressed by… - Problem with school

Not at all---------------------------------Very


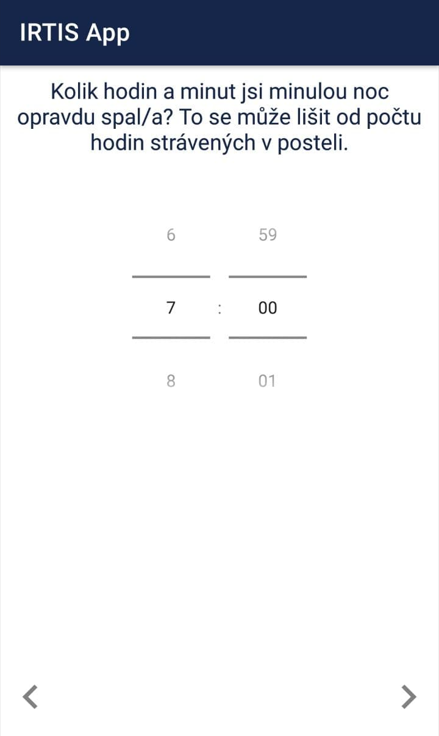

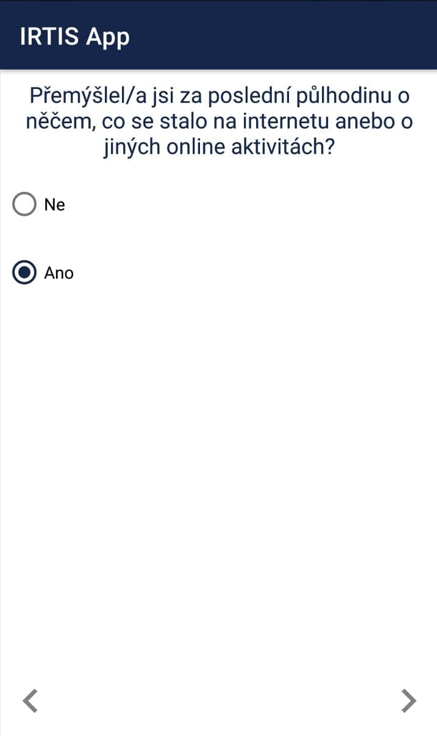


English caption:

IRTIS App

In the last half an hour, were you thinking about something that happened on the internet or other online activities?

- No
- Yes

English caption:

IRTIS App

How many hours and minutes did you actually sleep last night? (This may differ from the number of hours spent in bed)


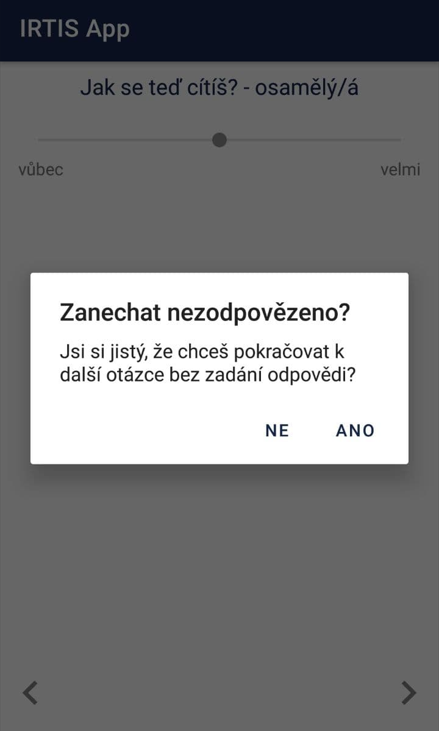

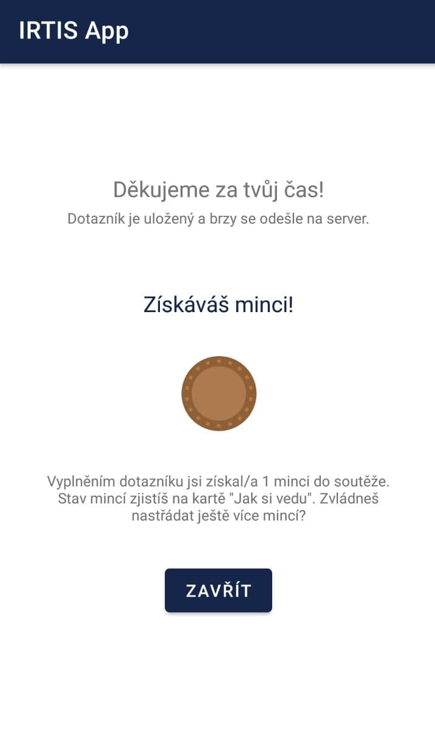


English caption:

IRTIS App

Thank you for your time!

The survey will be saved and sent to the server soon.

You gain a coin!

By filling out the survey you got 1 coin to the competition. You can find your coins on the My Progress section. Can you gain even more coins?

Close

Foreground:

Leave unanswered?

Are you sure you want to proceed to the next question without answering?

NO YES

Background:

How are you feeling right now? – lonely

Not at all -------------------------------- very


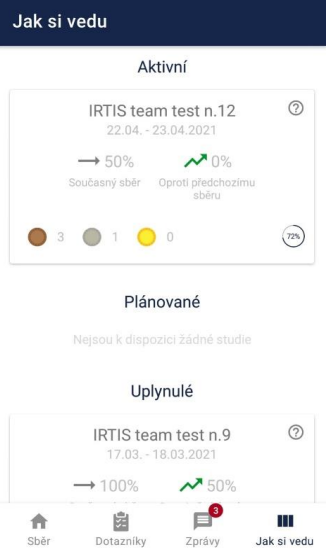


English caption:

My Progress

Active

IRTIS team test n. 12

22.04 – 23.04.2021

50% 0%

Current burst In comparison with previous burst

Number of coins


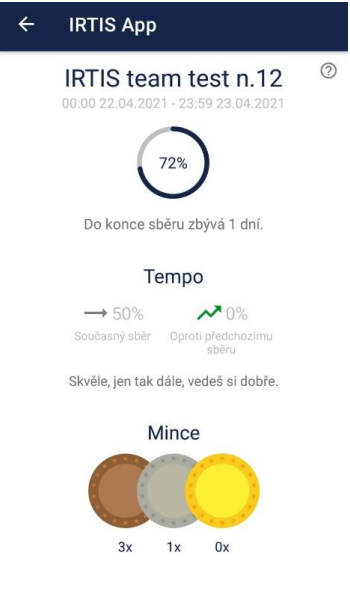


English caption:

IRTIS App

IRTIS team test n. 12

22.04 – 23.04.2021

72%

1 day left until the end of burst

Pace

50% 0%

Current burst In comparison with previous burst

Great! Keep going, you´re doing well.

Coins
