# Supplementary material for: Researching the Links Between Smartphone Behavior and Adolescent Well-being With the FUTURE-WP4 (Modeling the Future: Understanding the Impact of Technology on Adolescent’s Well-being Work Package 4) Project: Protocol for an Ecological Momentary Assessment Study
Source: JMIR Res Protoc. 2022 Mar 8;11(3):e35984. doi: 10.2196/35984 (PMC8941440; doi:10.2196/35984)
Supplement: Multimedia Appendix 4 [file resprot_v11i3e35984_app4.pdf]

GAČR

GRANTOVÁ AGENTURA ČESKÉ REPUBLIKY

Project Proposal Reviewer Form of 19-27828X:

Name of the Applicant: prof. PhDr. David Šmahel, Ph.D.
Project Title: Pohled do budoucnosti: Porozumění vlivu technologií na “well-being” adolescentů

Quality of the project proposal and project team:

Quality of the project proposal :
This is a high-calibre and forward-looking innovative project that investigates the influence and effects of the use of technologies on adolescents (formative years of 11-18) on the reality of their lives. This research aims to investigate physical, psychological and social factors in a coherent and integrative way and develops new methods of investigation in the field of artificial intelligence/machine learning. In analytical procedures, which are developed as models or prototypes, both information about the complexity of the effects is to be gained and statements about future effects are to be made. This is not speculative research, but a productive implementation and application of newer approaches to integrative research, such as those already developed across disciplines in diversity research. The project sensibly combines methods of media science, psychology and health care. In addition, the project expands the research spectrum of previous studies and closes an important research gap by linking data analysis, short-term and long-term surveys and experimental methods of artificial intelligence. These methods are elaborate and up-to-date with regard to the technological developments that have a great influence on the well-being of adolescents in particular. The choice of the target group is therefore well founded, also to make statements about future effects of these technological implications on our lives.
Preparedness of the project proposal:
The project will apply and interrelate models of Media Theory, Behavior Theory and Ecological Theory to investigate psycho-social and environmental effects of technology usage on adolescents and impact on behavior and quality of life. Departing from existing methods the project proposes an integrative framework that builds on data analysis, questionare and real time data collection through artificial intelligence (machine learning software) with the aim to comprehensively examine correlations, direct, indirect and reciprocal effects and how these have impact on each other. For this reason, the project employs researchers from Faculty of Social Studies and Faculty of Informatics (Department of Machine Learning and Data Processing) at Masaryk U. The tools complement one another and will allow to understand cross-relations, contextual effects, risks and benefits that have effect on the well-being of adolescents' usage of technology. The overall aim is to develop a new contextual framework that integrates perspectives and research tools and methods form diverse disciplinary and research fields. The project proposes moderate conference participations (12 conferences per year for 12 researchers). The work plan is tight and focused, majority of work is carried out at the Czech institution, where also most meetings and consultations with advisory board shall take place. The work areas are divided into defined work packages with different tasks and a clear structure, the transfer of knowledge and interconnections between the work tasks are ensured by personnel, and the respective use of methods and technological instruments is ensured. In view of the complexity and data volumes, the time frame is appropriate, the work programme is goal-oriented and geared towards the publication of essays, the publications are realistically dimensioned (21 articles), and a grant application by the applicant for ERC project is proposed at the very end respectively after completion of project.
Co-applicant and other members of the research team:
The research team consists of 12 researchers and is well-balanced between research leaders and post-docs and students. Work packages involve collaboration of younger researchers. In this respect, the professional quality is guaranteed and a balanced promotion of young scholars and students takes place. The technical, administrative, management and legal aspects of the project work are also adequately represented in the composition of the team.

Involvement of students and postdocs in the project:
see above (research team) The research team consists of 12 researchers and is well-balanced between research leaders and post-docs and students. Work packages involve collaboration of younger researchers and support promotion of young scholars and students.
Strengths/weaknesses of the project proposal and project:
The project will implement and prove a new integrative theory: this approach makes an important contribution to understanding the complexity of impacts of technology, setting the standard for future research. The distribution of workload/work packages ensures the operationality of integrative methods and knowledge transfer in interdisciplinary research. All members of the research team are excellently qualified according to their tasks and by carrying out this project they acquire new, cutting-edge expertise in integrative research, which enhances the international reputation of the institution and will have significant impact on the research landscape as a whole in the long term.
Appropriateness and justification of the financial costs:

Qualifications of the applicant:

The applicant:
The applicant is a highly ranked Professor having outstanding expertise as scholar and researcher in digtial technologies and social psychology with focus on youth studies. He has gained international reputation as leading researcher in his field and has widely published on the impact of digital media on health. He is recipient of significant number of prizes and has substantial experience as project leader/prinicipal investigator. Applicant has impressive record of attracting research funding and has managed large projects and respective teams. One ongoing research project that terminates in 2020 has no thematic relation to the EXPRO grant application. There is also no confilct regarding the 50 % workload that the applicant allocates to the proposed project.
Strengths/weaknesses of the applicant
The applicant has an international network of research activities in the area of the research project and was involved in various functions in research projects relevant to the topic and has initiated and managed numerous projects himself. The applicant's application shows that he has the technical competences, but also the skills of project and personnel management, to successfully manage a project of this size over a period of 5 years and to complete it with the corresponding outputs. Impact and outreach are considered from the outset, as are ethical and legal aspects of the project.

GAČR

GRANTOVÁ AGENTURA ČESKÉ REPUBLIKY

Project Proposal Reviewer Form of 19-27828X:

Name of the Applicant: prof. PhDr. David Šmahel, Ph.D.
Project Title: Pohled do budoucnosti: Porozumění vlivu technologií na “well-being” adolescentů

Quality of the project proposal and project team:

Quality of the project proposal :
This is a well though out project, in line with previous research, within a network of scholars who already work together. Its originality stems from the combination of objects and methods, rather than the influence of smartphone on well being (a topic which have coverage elsewhere as well). It addresses a socially useful problem (the positive and negative impact of technology on teen agers' well being), with methodological tools that are both adequate to the goals pursued (longitudinal and experimental surveys) and rather innovative (eye-tracking observation, automatic detection and learning designing), with due consideration for ethical issues. The project is carefully drafted, exhaustive, structured and organized in several work package (each aiming to deliver a specific outcome), it answers every questions that could possibly be raised about aims and methods, results expected, etc. it is not only empirically-based but also theory driven. Risks are listed and hedging solutions proposed. Relationship between variables, explanatory factors and drivers are modelled on a very telling graph. Possible breakthrough may come from a focus on positive impact, rather than negative effects; concern's for online and offline behaviour altogether; the respective explanatory power of emotional factors and personality traits, instead of confining the analysis to the usual suspects (demographics); prediction and not only observation. Overall, such advancement will contribute to international research on an equal footing with outcomes disseminated from well-established research centres (such as Stony Brooke).
Preparedness of the project proposal:
The proposal does not come out of the blue: it has been obviously prepared by meetings with local and foreign partners (like EU Kids Online, a 33 European countries research network) belonging to different disciplines. Aims are engrained in the explicitly formulated research questions (how to combine short term and long term, positive and negative, direct and indirect effects on adolescents' wellbeing) and the way to answer them (to capture subjective teen-agers' activity with the help of artificial intelligence, and the equal tracking of cognitive and affective effects altogether; software to anonymize respondents, which would be quite an innovation for colleagues). The number of variables that may explain respondents' behaviour is quite large. There is an impressive reflection on ethical issues, noteworthy as a quest for no-invasive methodologies. Depletion rates of panels of respondents are estimated, and alternative solutions imagined. Circularity is anticipated and possibly prevented. Dissemination is planned with some realism (about the length and risks entailed all along the publication process), although the number of articles that may come out seem overtly optimistic in such a span of time.
Co-applicant and other members of the research team:
The team is solid and cosmopolitan (with three non Czechs), and the project investigators will be accommodated by the Department of Machine Learning and Data Processing at the Faculty of Informatics as well as the Institute for Research on Children, Youth, and Family of the Faculty of Social Studies, Masaryk University. Connection with UCLA is ancient and maintained, and this is a promise of international attractiveness. The team is split into several subteam according to a sound division of labour, each knowing its specific agenda. Details abound about the complementarity of team members' skills. Each commits himself or herself to the assigned task, and says what percentage of his or her time will be devoted to it. Publication records are quite satisfactory.
Involvement of students and postdocs in the project:
Three postdocs will work on the project, with details about their respective work plan. No other student seems to be involved at that stage.

Strengths/weaknesses of the project proposal and project:
Most strengths, few weaknesses. This proposal reaches the level of expectation of ERC committees. Instead of caveats the draft has some lacunas, like the design of the machine learning software. Details about experiments that are not fully explicit can nonetheless be deducted from the critics made against previous experiences elsewhere. To be exhaustive, one can ask a killer question, without any mischievous intent though: why is it so important to work on the impact of new technologies on adolescents' wellbeing within the social sciences? At any rate the answer is implicit if not fully developed in the draft, again: health scientists would not include emotional states in their surveys.
Appropriateness and justification of the financial costs:

Qualifications of the applicant:

The applicant:
David Smahel has multiple training in computer sciences, psychology, and sociology at Masaryk University (with a 2-month mobility at UCLA). He became full professor 11yrs after completion of his PHD. He is experienced in cross-national surveys, and granted projects. Editor of a specialized academic Journal and advisor to UNICEF, and member of two international epistemic networks, he authored an important book in 2011, and coedited another one in 2018. Several articles were published in high impact factor Journals, some co-authored with US scholars like two developmental psychologists (Subrahmanyam and Greenfield). Most concern health issues and the development of cognitive skills and emotional states by children.
Strengths/weaknesses of the applicant
Assets are: Multidisciplinary training, focus on the relationship between technology and wellbeing, participation to cross national surveys, rigour and methodological refinement. Lacunas: trained and hired in the same university, one visiting stay only (at UCLA). Overoptimistic about the number of publications that will come out of the project once completed.

GAČR
GRANTOVÁ AGENTURA ČESKÉ REPUBLIKY

Project Proposal Reviewer Form of 19-27828X:

Name of the Applicant: prof. PhDr. David Šmahel, Ph.D.
Project Title: Pohled do budoucnosti: Porozumění vlivu technologií na “well-being” adolescentů

Quality of the project proposal and project team:

Quality of the project proposal :
The project’s overall goal is to develop a comprehensive integrated evidence-based theory depicting the complex impacts of technology usage on physical, psychological, and social well-being of adolescents from both the short and long-term perspectives. The topic and aims of the project are relevant, scientifically important, and the expected results of the project are significant. The expected international cooperation is very high thanks to the strong connections of the IP with international researchers in the area. Nevertheless, the main innovation of the project is methodological.
Preparedness of the project proposal:
The aims of the project are well defined, relevant, and the expected results of the project proposal are the publication of 21 articles in professional journals (impacted), 6 of them in the best journals in the field. The proposal is well-written and prepared. The interconnectedness of the 5 WPs is very well explained. The applicant should better highlight the main contributions of this project to the existing literature.
Co-applicant and other members of the research team:
The team members are part of the Interdisciplinary Research Team on the Internet and Society (IRTIS) and the Department of Machine Learning and Data Processing. The professional skills of the research team are very adequate to carry out the project. The workplace conditions and the state of readiness of the workplace are also adequate to carry out the project.
Involvement of students and postdocs in the project:
3 postdocs and 4 students will be involved in the project. Their contribution to the project is well described and their involvement will be beneficial for both the project and the postdocs and students.
Strengths/weaknesses of the project proposal and project:
Strengths: - Relevance of the topic of the project proposal. - Definition of the objectives. - International cooperation, strong connections of the IP with international researchers in the area. - Team members. Weaknesses: - Relevance of the expected results, since the main innovation of the project is methodological. - The expected number of papers published in the best journals in the field is not very high.
Appropriateness and justification of the financial costs:

Qualifications of the applicant:

The applicant:
The applicant’s qualification and ability to carry out the project are very high. He has participated in numerous invited lectures, he has received several prizes and he has participated in numerous relevant research projects. He has He has also supervised several PhD students and has contributed to the development of his scientific field in the international context. However, the academic papers of the applicant are not published in the best journals in the field.
Strengths/weaknesses of the applicant
Strengths: - Participation in relevant research projects. - International research and work experience. - Publication activity. Weaknesses: - The academic papers of the applicant are not published in the best journals in his field.

Project Proposal Reviewer Form of 19-27828X:

Name of the Applicant: prof. PhDr. David Šmahel, Ph.D.
Project Title: Pohled do budoucnosti: Porozumění vlivu technologií na “well-being” adolescentů

Quality of the project proposal and project team:

Quality of the project proposal :
The proposal is high interest combining aims (develop theory about the impacts of technology usage on physical, psychological and social wellbeing), the target group (adolescents aged 11 to 18) and some of the methodologies developed (software using artificial intelligence). This combination makes it more original, potentially contributing to orientate new technologies. In relation to the expected international cooperation, an in addition to the involvement of external members in a sounded advisory board, formal participation in research is foreseen from several reputed scholars from EUKO network (EU Kids Online), mainly in WP1. The project seems to have high potential innovativeness as combining short and long-term effects, which would facilitate better modelling future impacts of technology on well-being. It is of special interest the inclusion of the longitudinal perspective in the analysis of long-term effects. In this sense, the project may imply a valuable progress in relation to current methodological approaches as well knowledge base.
Preparedness of the project proposal:
The project is very well designed in terms of WP structure, highly consistent. Each WP is very well developed, including all of most of key aspects in terms of objectives, specific methodology, ethical issues, risk scenario, deliverables and milestones, innovativeness and interconnections with other WPs. In addition, time schedule is sufficiently consistent and clear in the Gantt chart, although it would be better explained. A very useful global risks analysis, in addition to some statements for each of the WP, is done, trying to prevent potential imbalances in relation to what has been foreseen and is expected. Thus, it includes some clarifying thoughts on personnel risks, project outputs, project schedule and tender for longitudinal survey. In this risk analysis applicant recognises that project expected outputs is ambitious. In particular, it is foreseen a total of 21 papers in journals with IF, which seems, certainly, a very high number of outputs. The distribution of number of papers per WP is stated. However, after the number of papers, more indications, even brief or approximative, about contents and journals in each WP, would be welcome. A second type of outputs will be presentations at conferences of a high prestige. Although it is mentioned in some of the WP, a more systematic explanation on the foreseen attendances would be welcome. A third output will be an ERC project submission. Some additional information about this foreseen submission would be welcome, and how the present project would “feed” that ERC proposal.
Co-applicant and other members of the research team:
Research team seems to be sufficiently experienced with the professional needed skills in each of the WP participating. A clear distribution of tasks is stated. From some general statements it seems that team members will be fully involved in the elaboration and publication of results. Nevertheless, more detailed information about individual CVs would be welcome, for example date of PhD and title and dates of projects in which S/he has participated. This is especially important to better know about the career and experience of, for example, the three postdocs who are team members (in spite of the useful indications already gave). On the other hand, it is not sufficiently explained how to avoid overload for those members that should lead one highly time demanding WP, and collaborate at the same time in others WP or projects (also considering a dedication of 0,5 for all of team members). It seems to have a risk that in some periods those team members will be concentrate in their leaded WP, so it may negatively affect the needed contributions in others WP. Applicant, as a main responsible, should be aware to efficiently manage this potential risk. One way to ensure collaborations are the juniors

researches, who may be more specialised and focused in some tasks (or WP), with full or part time dedication. But it would also be risky to reside a main contributor workload in junior researchers (even considering they will be in charge of more "mechanical" tasks, such as data coding and cleaning).

Involvement of students and postdocs in the project:

Three out seven of team members are postdocs (also 0,5 of dedication each of them), with important responsibilities. One of them will lead one WP4. From the short indications it seems she is experienced enough to successfully manage that tasks. Additionally, a total of five junior researchers will be hired to support senior researchers. A total of four students will also participate, three of them from the beginning of the project. Each of them will contribute with 1.000 hours (200 per year) (except one of them, with 800 hours). The positive aspect is to involve students in practical aspects of the research, which is a learning process for them. Nevertheless, it should be avoided the risk to reside too much work and/or responsibilities in them (for example, preparing literature review, which seems, in principle, that should be a task for a postdoc or a senior research).

Strengths/weaknesses of the project proposal and project:

Strengths • Very relevant topic in the frame of societal changes • Well designed in terms of aims and objectives, as well as conceptual frame • Accurate methodology, which will ensure good and relevant results • Consistent structure of WP, with clear explanations, when necessary, in terms of objectives, specific methodology, ethical issues, risk scenario, deliverables and milestones, innovativeness and interconnections with other WPs. • Apparently experienced enough research team • Outputs intended to be published in top journals (clear specification of 6 of the contributions) Weaknesses • Not fully convincing ambitious publication plan, which needs clearest explanations about its real feasibility • Not fully explanations are given about the experience of some team members • Not fully convincing explanations (even through the risk analysis) on how to cope with some distribution of work load. In particular, some doubts emerge from the need for high concentration leading some of the WP, and at the same time having to collaborate in other WP, which may derive in that important parts of the work reside mostly on junior researchers of even students. Globally speaking, strengths are much more relevant than the weaknesses, with the final result of an excellent and consistent proposal.

Appropriateness and justification of the financial costs:

Material cost seems to be overestimated, or at least explanations don't seem to be sufficient. In travel cost it would be necessary better justify the high number of attendances to conferences (12 attendances per year) and the apparently high unitary cost per each of it. Meetings with advisory members seems also overestimated, and perhaps it would be explored less frequency or the use of videoconferences facilities, reducing the high cost. Explanations about the cost for "Sampling and recruiting of respondents", for WP4, included in "other services", would be welcome.

Qualifications of the applicant:

The applicant:

Dr Smahel has a career of specialisation since the last 90's. His has an excellent background in relation the project, combining Informatics (M.Sc), Sociology (B.A) and Psychology (M.A), with a PhD in Social Psychology (2003). After his intensive career he leads the Interdisciplinary Research Team on Internet and Society, an excellent frame for the development of this project. He has mentored five PhD students and two postdocs. During the last 5-6 years he has developed an intensive activity participating or leading research projects, some of them directly related to the present one. All these activities as well as his outstanding selected publications show he is very well positioned to carry out this project, since he has very good professional skills (complemented with those form some of the remainder team members), very good theoretical and conceptual background, and experience managing research teams.

Strengths/weaknesses of the applicant

Strengths • Consistent background, combining Informatics, Sociology and Psychology, which may allow him to better conceptualise research problems and conceive and design outstanding research projects; • Sounded selected publications, published in high top journals of editorials; • Experience managing and conducting research projects, some of them of international scope; • Some international experience, collaborating in several research projects. Weaknesses: • Although Applicant participate in some international projects (such as EU Kids Online – EUKO) and he is in touch with reputed scholars (for example, those from Advisory board), the resulting

networking doesn’t seem to be especially strong or with especially relevant results. For example, top publications don’t use to involve foreign scholars. • In spite of the intense career, the most important in terms of outputs production and projects seems to be a bit short, limited to the last 5-6 years.

GRANTOVÁ AGENTURA ČESKÉ REPUBLIKY

Project Proposal Reviewer Form of 19-27828X:

Name of the Applicant: prof. PhDr. David Šmahel, Ph.D.
Project Title: Pohled do budoucnosti: Porozumění vlivu technologií na “well-being” adolescentů

Quality of the project proposal :

Originality, scientific importance, project prospects and expected benefits of the project for research:
The main purpose of the study is to examine the associations between technology usage and dimensions of well being among adolescents. The author(s) has presented a well-written project proposal. In my opinion, the longitudinal aspects of the study will display the long term effects of technology usage in well-being.
Preparedness of the project proposal, methodology, concept, project aim and proposed deliverables:
There is a lack of studies as to the long term effects of technology usage in psychopathology and well-being. Overall, the research proposal has several strengths in terms of aims, methods, process and theoretical background.
Co-applicant and other members of the research team:
I think that the research team is qualified so as to conduct this research project. The publication level of research team is praiseworthy.

Qualifications of the applicant:

The applicant:
I think that the researchers have many potentials to conduct this project properly. The researchers have reported that 21 articles are planned to be continuously submitted throughout the whole project. Additionally, they reported that these articles are planned to submitted to journals with impact factors. This effort will make a significant contribution to the sharing of project results with the world of science and other researchers.

Overall commentary on the project proposal:

Strengths of the project proposal:
One of the strengths of the project proposal is method. The researchers will declare a theory based on empirical data from existing research, 3-wave longitudinal study, series of experimental studies and data collections with measures. Although there are many studies in relevant literature examining the associations between technology usage (in a simple dimension, such as internet addiction, problematic internet use, facebook addiction etc.) and well-being (only single dimension, for instance subjective well-being, life satisfaction etc.) with a cross-sectional design, the effects of technology use on multidimensional well-being has not been addressed in accordance with the time-based change. This is the most important aspects of the research project.
Weaknesses of the project proposal:
Research team will conduct this study with quantitative research approach.
General comments:
The research project has many strengths. When I read this project proposal, I have a very strong belief that the research team can carry out this project.

GAČR
GRANTOVÁ AGENTURA ČESKÉ REPUBLIKY

Project Proposal Reviewer Form of 19-27828X:

Name of the Applicant: prof. PhDr. David Šmahel, Ph.D.
Project Title: Pohled do budoucnosti: Porozumění vlivu technologií na “well-being” adolescentů

Quality of the project proposal :

Originality, scientific importance, project prospects and expected benefits of the project for research:
This is an important and ambitious proposal involving a well qualified Czech researcher and an international team. Adolescent use of the internet is a fast developing area and important area. Sonia Livingstone at LSE in London has led much of this work. I was pleased to see that she was on the advisory board. Five work programmes, four involved in collecting data are planned over a five year period. The challenge in this project will be to keep up with the fast developing changes. If papers/outcome are not published as the project develops, the findings could be out of date before the research is finished. I do not know of any current research that is looking at different aspects of well-being related to internet usage.
Preparedness of the project proposal, methodology, concept, project aim and proposed deliverables:
Professor Smahel has prepared well for this proposal. His earlier research suggests that he is on top of the complex methodology and should be able to meet his targets. I like the range of data that he will use: The use of the EU Kids Online network; a longitudinal study of Czech adolescents and carers (as he notes attrition is always a problem in this sort of data) and experimental studies. Developing new research tools (WP4) will also be useful and looking towards a new theory of media effect on well-being is important. He divides core internet activities into 'use of different information' and 'communication technologies'. This may prove simplistic as technologies develop. I was not sure where 'gaming activities' fitted in but from Dr Smahel's CV, I see he has written papers about this. Patterns of usage is also be important. I was pleased to see that ethical issues were considered at every stage.
Co-applicant and other members of the research team:
Members of the research team appear well qualified and should contribute the the success of the project..

Qualifications of the applicant:

The applicant:
Professor Smahel appears well qualified to lead with project. This is an important developing area of research and he could find himself leading international authority

Overall commentary on the project proposal:

Strengths of the project proposal:
This is a highly topical and important area of research which has grown in importance as adolescents have become more and more attached to their internet activities. Although information is emerging, we still do not know how different aspects of internet use and different usage affects different aspects of adolescent well-being.
Weaknesses of the project proposal:
The area is developing fast and Professor Smahel could find, as technology develops, that his findings are out of date before they are published. This however is not a reason to reject the proposal. The EU study may already be dated. The division of internet usage into 'information activities' and 'communication technologies' is a bit simplistic. I was not sure where 'gaming activities' came in. Thee are also a large number of variables to take

account of: age of young person; gender; usage; (in the Czech study) parental control etc. The longitudinal study will prove challenging because of drop out.

General comments:

This as I have mentioned is an ambitious but important project which I feel should go ahead.

GRANTOVÁ AGENTURA ČESKÉ REPUBLIKY

Project Proposal Reviewer Form of 19-27828X:

Name of the Applicant: prof. PhDr. David Šmahel, Ph.D.
Project Title: Pohled do budoucnosti: Porozumění vlivu technologií na “well-being” adolescentů

Quality of the project proposal :

Originality, scientific importance, project prospects and expected benefits of the project for research:
The main objective of the proposal is to provide a comprehensive theory of the short and long-run impacts of technology on children and adolescent well-being driven by empirical evidence. Elements of this project are outstanding and deserves full support, given the fragmented state of the knowledge.
Preparedness of the project proposal, methodology, concept, project aim and proposed deliverables:
The proposal is very well prepared, the timeline is meaningful and concepts are worthy of support. However, there are elements that suffer from selection bias and are neither satisfactory nor useful to conduct the truly innovative WP3, WP4 and WP5 in my view. Moreover, WP2 does not offer a truly long-run analysis as it is a 3-year study. I advance examples on how to improve upon this in the Overall Commentary section.
Co-applicant and other members of the research team:
The team is build around the PI, his competence and ability to deliver, and both the project management and organisational charts denote clear division of labour, competence. The team seems to have been carefully put together and ready to conduct the different elements of the study.

Qualifications of the applicant:

The applicant:
The CV of the application is excellent and the fit to this project is impressive. The part of the proposal highligting risk and readiness show diligence and careful thinking about the potential issues arising.

Overall commentary on the project proposal:

Strengths of the project proposal:
The strenghts of the project rest on the amin objective, the devise of a comprehensive theoretical framework, the team and the innovative studies proposed in WP3 and WP4, which will come together in WP5. I believe that WP3 and WP4 can be expanded, at the expense of WP2, to offer truly new knowledge together with an exploratory analysis of existing EU Kids datasets. I offer more details on this in the next section.
Weaknesses of the project proposal:
The current proposal suffers from 2 fundamental weaknesses in my view. First, the analysis of existing datasets (WP1) as currently proposed will not deliver satisfactory causal effects. Second, the proposed longitudinal survey (WP2) will suffer from the same weaknesses (inability to identify causal effects given) and will not offer much regarding long-term effects (3 years does not capture long- term). Existing surveys and proposed new ones should be combined with research design attempting at identifying plausibly exogenous changes in exposure to social media at individual or area level. My general evaluation of the current knowledge -- reviewed in the proposal -- is that it tends to confirm priors. The empirical strategies adopted do not seem to in disentangling simple observed differences from causal effects. This subject is too important to let confirmation bias, and researcher degrees of freedom, sneak-in. I would hope that a well-funded project at this stage would focus on tackling the lack of causal analysis head on, even when using existing or ad-hoc surveys. Although WP1 mention

the word “association” instead of causal effects, the theoretical framework and conceptual model (Fig 1) refers to effects (hence causal paths). This confusion is not useful. While I agree that WP3 and WP4 can provide powerful analyses in the direction of the discovery of causal effects, neither WP1and WP2 offer any strategy that will enable the researchers to say anything causal. The existent EU Kids datasets will only recover associations. Associations between, say, (subjective evaluations of) hours spent on social media and (subjective evaluations of) lack of sleep does not represent a causal effect that goes from one to the other. For instance, underlying unobserved conditions of anxiety, that might not be picked up by any control variable, will confound the analysis. There are a number of potential confounders here that no datasets could satisfactorily account for. These issues are not mentioned by the applicants. This is not to say that the analysis of existing datasets proposed in WP1 is not informative, but it is more a preliminary work to WP3 and WP4 rather than a standalone contribution. This analysis is useful as preliminary. The longitudinal study proposed in WP3 is more problematic. The endeavour – as it is – does not convince me it represents good value for money. It does not address causality (observing the same individual over time addresses some concerns but will not solve reverse causality or simultaneity bias or other confounders) and it does not provide much in terms of long-run analysis either. 3 years is just not long-run enough. Other strategies that can identify long-run effects. For instance, why not combining different datasets and employing quasi-experimental design detect genuine long-run effects? For example, the applicants should spend some time identifying plausible exogenous variations in exposure to social media occurred at time t in area a, and then combine these changes to existing longitudinal surveys (such as the German Socio Economic Panel or the British Household Panel Study). This panel includes samples of children at time t in area a and enables researchers to compare their well-being N years after the exogenous change in time t, to other statistically similar children that were not exposed to the same change. As it stands, i.e., in the absence of rigorous research design, I would prefer this project to focus and expand WP3 and WP4, which I truly find promising and outstanding, rather than engage in the very complex task of longitudinal survey administration.

General comments:

I do believe this proposal includes elements of outstanding innovation and research. The good news is that I do think WP1, WP3, WP4 and WP5 can go ahead without WP2.
